# The use of reduced graphene oxide (rGO) as de-icing agent in engineered cementitious composites (ECC)

**DOI:** 10.1038/s41598-025-14327-y

**Published:** 2025-08-15

**Authors:** Hasan Erhan Yücel, Maciej Dutkıewıcz, Muhammed Dıkmen

**Affiliations:** 1 Department of Architecture, Faculty of Architecture, Hatay Mustafa Kemal University, Hatay, 31060 Turkey; 2 Faculty of Civil and Environmental Engineering and Architecture, Bydgoszcz University of Science and Technology, 85-796 Bydgoszcz, Poland; 3 Department of Civil Engineering, Vocational School of Technical Sciences, Kilis 7 Aralık University, Kilis, Turkey; 4 Nigde Ömer Halisdemir University, The Graduate School of Natural and Applied Sciences, Niğde, Turkey

**Keywords:** Rigid pavements, Engineered cementitious composites (ECC), Reduced graphene oxide (rGO), Electrical and heating properties, Engineering, Civil engineering

## Abstract

In this study, it is aimed to prevent traffic accidents caused by icing by producing an innovative concrete pavement that is both heatable and has high ductility in order to solve the ductility and icing problems encountered in rigid pavements used in highways. For this aim, rGO-ECCs were produced by adding different proportions of Reduced Graphene Oxide (rGO) to Engineered Cementitious Composites (ECC) known for their high ductility properties. The mechanical, electrical and heating properties of rGO-ECCs in different environments were investigated. Moreover, their energy efficiencies during deicing procedure were compared. Compression test was conducted to all rGO-ECC mixtures. In addition, two-pole conductivity test was performed to measure the resistivity values of rGO-ECCs. Furthermore, heating processes were carried out by applying a carbon-based conductive liquid coating to the surface of rGO-ECCs. Heating processes were applied at room conditions (22C°) and in a cold environment test room (-33C°). The energy efficiency was calculated by measuring the temperature changes of the samples with a thermal camera. TGA-DTA, FT-IR, XRD and SEM–EDX analyses were performed to determine the microstructural properties of rGO-ECCs. Compared to the control (0.0% rGO-ECC) sample, the compressive strength of the 0.6% rGO-ECC sample decreased by approximately 26% and was determined as 52 MPa. The resistivity value of the control sample, which was 4115KΩ.cm, decreased to 49KΩ.cm with the addition of 0.6% rGO-ECC and the conductivity increased. The energy efficiency for temperature change in room (22C°) and cold ambient (-33C°) conditions was calculated as 59.7% and 86.1%, respectively, and the energy efficiency for melting 6 cm of ice in a cold environment (-33C°) was calculated as 63.1%. Finally, a cost analysis was made for ECCs. As a result, it was concluded that rGO can be used as an effective de-icing agent in ECCs.

## Introduction

There are two types of pavements encountered on highways^1^. These are flexible and rigid pavements^2^. Flexible pavement consists of sub-base, base and bitumen binder coating layer^3^. Bitumen is a dark brown-black colored binding material obtained from crude oil^4^. Rigid pavement consists of cement binder coating and sub-base layer^5^. Rigid pavement is used in regions with heavy vehicle load effect and heavy traffic^6^. However, an important problem arises in rigid pavement. This problem is that the traditional concrete used in rigid pavement exhibits brittle behavior under the effect of load. The brittle behavior of traditional concrete causes cracks on rigid pavement. For this reason, the low bending ability of traditional concrete has revealed the need for a more ductile concrete^7^. In addition, in rigid pavement, the permanent ice layer that occurs on the roads due to cold weather and snowfall and cold weather throughout the season has endangered traffic flow. Traffic accidents have occurred as a result of icing and as a result of the accidents, there have been losses of property and life^8^. In order to eliminate the insufficient bending strength and crack formation in traditional concrete and the ice layer formed on the pavement, the production of a rigid superstructure with high bending performance and without ice formation is of vital importance. In order to produce this vital concrete, new materials must be added to the concrete matrix.

Traditional concrete is the most basic concrete produced using aggregate, cement and water. The most important problem of this concrete is that its bending strength is very low. Many types of fiber concrete have been produced to improve its bending ability and the bending strength of traditional concrete has been increased^9,10^. One of these developed fiber concretes is Engineered Cementitious Composites (ECC). Cement, fly ash, silica sand and polyvinyl alcohol (PVA) fiber have been used in the ECC mixture^11^.

Compared to traditional concretes, it has been observed that the bending strength and energy absorption performance of ECCs are much higher and high ductility values have been achieved. In addition, PVA fiber concrete has shown better results in the mixing of freezing and thawing events^12,13^. Thanks to ECC produced with PVA fiber, an average of 400 times more ductility has been provided to the rigid pavements that exhibits brittle behavior^14,15^.

Ploughing, salting and anti-freezing chemical materials have been used to remove ice from the roads caused by snowfall^16^. During the snow-ploughing process, due to the heavy weight of the snow-plough machines, cracks/breaks occur in the coating and the steel heads used for ploughing damage the pavement surfaces by scraping the top layer^17^. In addition, some chemical solvents such as salt and calcium acetate are used to prevent icing and to dissolve ice. The use of salt and chemicals causes a decrease in the strength of the pavement and cracks^18^. In road pavements, the freezing–thawing event is quite effective in damaging the pavement in a short time. The water entering the pores in the pavement undergoes a liquid–solid state change with the freezing–thawing event. The volume of the water undergoing a liquid–solid state change increases by approximately 10%. For this reason, the hydraulic pressure inside the pavement increases and damages the pavement by cracking it^19^. After freezing–thawing and shoveling, the service life is shortened and financial losses occur^20^. It has been observed that salting and chemical materials damage the pavement overlay in the long term^21,22^.

Additionally, studies have been conducted on directly heating the pavement or melting ice by heating conductive materials placed inside the pavement^23–25^. Metal powder, copper powder, carbon-based materials (graphite, carbon nanotube, graphene and its derivatives) were added to the concrete mixture for direct heating of the pavement and the conductivity properties of the concrete were increased^26–31^. In the studies using graphene, graphene oxide and reduced graphene oxide in the production of concrete, it was reported that the processability decreased as the graphene oxide ratio increased, and the 0.03% graphene oxide ratio showed the best improvement on the mechanical properties^32–35^. With the use of graphene at low rates such as 0.06%, a 15% increase in compressive strength and an 18% increase in flexural strength were achieved and it was reported that graphene used at high rates negatively affected the hydration process and the chemical interaction between gel crystals^36–39^. 0%, 0.01%, 0.02%, 0.03%, 0.04% and 0.05% rGO were added to the cement paste by weight and the results obtained show that significant increases of approximately 27% and 38% in compressive strength were obtained by adding 0.04% rGO and 0.05% rGO to the cement matrix, respectively^40^. The effects of adding rGO to the cement mixtures at the rates of 0.04%, 0.08% and 0.12% on micromorphology, hydration and strength were investigated. When the results were examined, the best values were determined in the mixtures containing 0.08% rGO. When the 28-day compression and flexural test results were compared, it was reported that the mixture containing 0.08% rGO increased by 30.3% and 24.3%, respectively, compared to the control mixture. It was observed that the void ratio decreased significantly with the addition of rGO to the mixture^41^. In another study where rGO was used in cement mortars, the addition of 0.02% rGO by weight to the cement increased the 7 and 28-day flexural strength of the mortar mixture by 70% and 23%, respectively^42^. The heating of the pavement was provided by conductive mesh (copper or steel wires) placed as resistors in the rigid pavement overlay^43–47^. According to the experimental results obtained in the study on the use of graphene oxide in ECC, significant increases were obtained in compressive strength, direct tensile strength, flexural strength and elasticity modulus by adding 0.05% GO(Graphene Oxide) and 1–1.5% PVA fiber. These increases were found to be 30%, 35%, 49% and 33.9%, respectively. It was observed that positive results could be obtained for the construction sector by adding 0.05% GO to ECC^48^. It has been observed that the use of graphene and its allotropes in ECC contributes to the increase of the high ductility and strength properties of ECC^49–53^. In addition, when graphene and its derivatives are added to the ECC matrix, the resistivity values of the insulating ECC are reduced and conductivity properties are gained, thus increasing the conductivity values^54–59^. Waste quarry dust (WQD) was used in ECC at 10%, 20% and 30% rates instead of cement. Using 20% WQD instead of cement increased the compressive strength by 14.2%^60^. In ECC, stone processing waste (SPW) was used in the ratios of 10%, 20% and 30% instead of sand. Using 20% SPW instead of sand increased the compressive strength by 8.5%. Using 20% of physically treated crumb rubber (CR) increased the compressive strength by 15.2%^61^. Ceramic powder (CP) was used in engineering cementitious composites (ECC) at 20%, 40%, 60% and 80% rates. The effect of using magnetized water (MW) instead of tap water (TW) was measured in ECC mixtures containing 0% and 20% CP. A 20% improvement in ECC workability was observed when CP or MW was used. It was observed that the compressive strength of ECC decreased with the increase in CP content between 20 and 80%^62^. In many studies, mechanical and physical properties have been investigated, but no study has been conducted on conductivity and thermal performance (heating and defrosting). With this study, the properties of rGO-ECC that were not mentioned by other studies were investigated and an important gap was filled. In a study conducted with copper-coated fibers and rGO, the conductivity and deicing performance of concrete produced in the dimensions of 30 × 30x15cm were investigated. The 28-day compressive strength was determined as 32.6 MPa. A 35% decrease in compressive strength was observed compared to the control sample. 15 cm snow melting experiments were conducted by applying 108 V. As a result of snow melting energy efficiency calculations, energy consumption decreased by 16.80%^63^. According to this study, it is seen that the study conducted is more advantageous in terms of compressive strength, energy consumed and energy efficiency obtained. Snow melting performances were investigated with thermal conductive concrete containing copper powder and silicon carbide. Samples with copper powder and silicon carbide added melted ice 2 h earlier than the control sample. The highest compressive strength was determined as 50 MPa. The best ice melting performance was 89% of the ice after 360 min^64^. According to this study, it is seen that the study performed is more advantageous in terms of energy efficiency and ice melting performance. Recent studies have investigated the mechanical and electrical behaviors of concretes with various conductive and nanomaterial additions. Although these studies reported improvements in mechanics and conductivity, the energy efficiency, mechanical, heating and de-icing performances considered in this study seem to provide advantages.

In this study, it is aimed to find a solution to the insufficient ductility and icing problems encountered in rigid pavement overlays by adding rGO into the ECC matrix.rGO-ECC was produced using three different rGO ratios as 0.0% rGO (Control), 0.4% rGO and 0.6% rGO. Compressive test was performed to determine the compressive strength of the rGO-ECC mixtures. In addition, XRD, TGA-DTA, FT-IR and SEM–EDX analysis results will be examined to see the microstructural features. Moreover, a two-pole conductivity test will be performed to control the conductivity and resistivity values. After this stage, heating experiments were performed under different environmental conditions to monitor the heating performances of rGO-ECC. Finally, cost analysis and comparison of ECC and rGO-ECC were made using 2024 prices. For the experimental method, mechanical, electrical, thermal and defrosting experiments were carried out with rGO-ECC samples. It was determined that the compressive strength decreased due to the increasing rGO rates but within the limits for rigid pavements. As a result of two-pole conductivity tests, it was seen that the conductivity of rGO-ECC increased but was not sufficient to heat the samples. It was determined that higher rates of rGO should be used to heat the samples. Instead of increasing the rGO rate, a carbon-based conductive coating was developed to eliminate this problem more economically and the heating process was carried out by coating the surface of the samples. Heating experiments were performed at room temperature (22C°), heating experiments in a cold environment rehearsal room (-33C°) and 6 cm artificial ice melting experiments in a cold environment rehearsal room (-33C°). The compressive strength of the control (0.0% rGO-ECC) sample was determined as 70.1 MPa, and the compressive strength of the 0.6% rGO-ECC sample was determined as 52.19 MPa. Although approximately 26% loss in compressive strength was observed, this value is 41% more than the limit value. The resistivity value of the control sample, which was 4115 KΩ.cm, decreased to 49 KΩ.cm with the addition of 0.6% rGO-ECC and its conductivity increased. The energy efficiency for temperature change at room temperature (22C°) and cold ambient temperature (-33C°) was calculated as 59.7% and 86.1%, respectively, and the energy efficiency for melting 6 cm ice in a cold environment (-33C°) was calculated as 63.1%. As a result of these experiments, heating, energy efficiency and ice melting performances were determined and compared with the control sample. Although many studies have investigated the mechanical properties of ECC, very few studies have conducted experiments, especially electrical conductivity, heating and defrosting under different ambient conditions. When ECC is modified and enhanced with a key material such as rGO, a multifunctional ECC is produced. By adding rGO, it can fill an important gap for an ECC rigid pavement coating with high strength, conductivity and de-icing performance. Additionally, rGO-ECC was be able to melt the ice on it by itself with very low energy consumption. The resistivity values of the concrete were reduced thanks to the conductivity of rGO. Since conductivity and resistivity are inversely proportional, the conductivity of ECC was increased. ECC with increased conductivity by using rGO was usable in solving the icing problem. The aim of the study is to investigate the thermal and electrical behavior of rGO and ECC composites under different environmental conditions. In addition, it is aimed to prevent accidents caused by icing on highways by providing de-icing properties in rGO-ECC. Although some studies have investigated the properties of ECC, they have limitedly studied the combined effects on both thermal and electrical performance using reduced graphene oxide (rGO) on heating and defrosting performances under different ambient conditions. This study aimed to fill this gap.

## Experimental studies

### Materials and preparation of samples

**Fig. 1:**
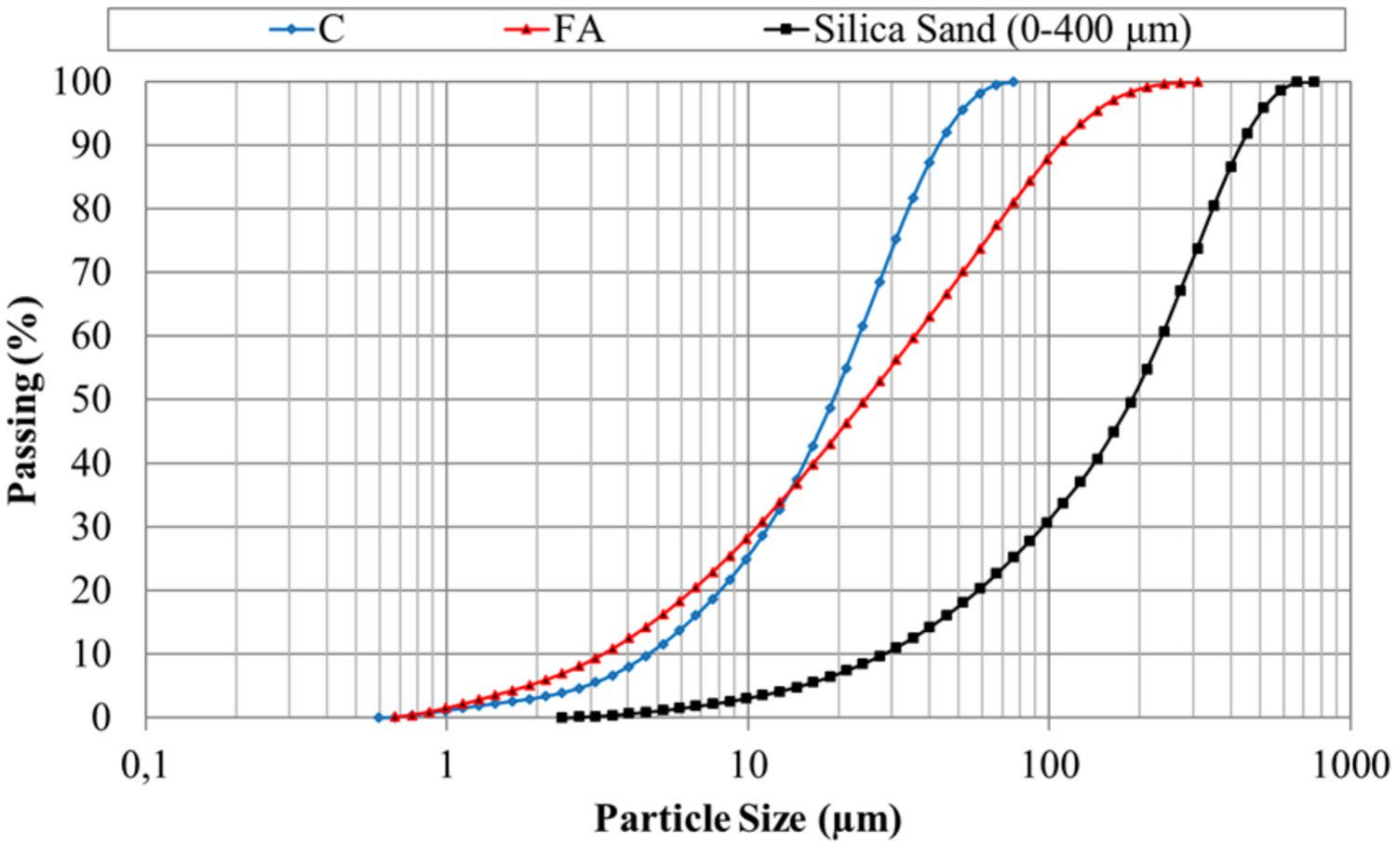
Grain size distributions of cement, fly ash and silica sand.

**Fig. 2:**
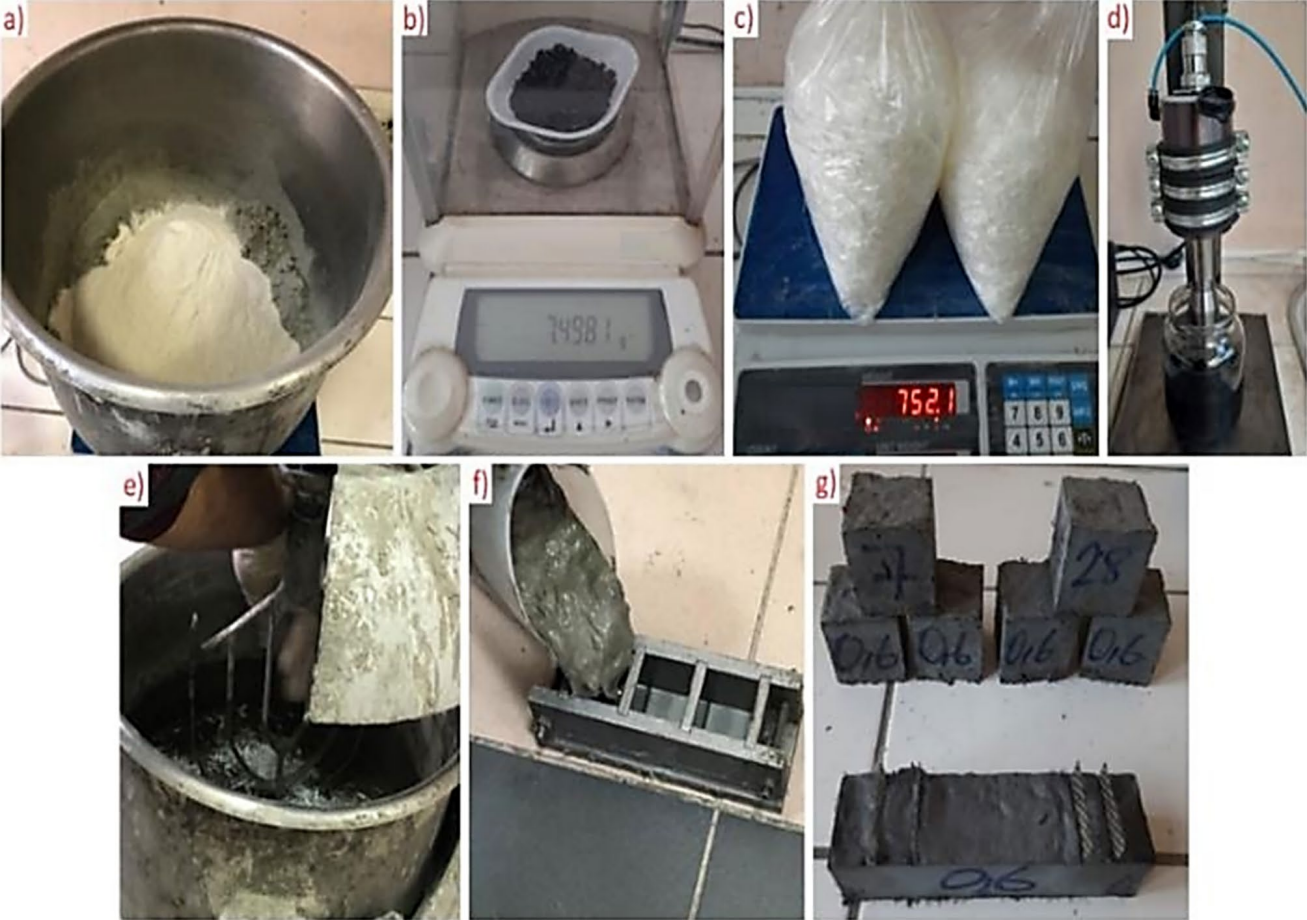
(**a**) Weighing of binders and silica sand, (**b**) Weighing of rGO with precision balance, (**c**) Weighing of PVA fiber, (**d**) Sonication process for rGO homogenization, (e) Mixing with mixer, (**f**) Placing in mold, (**g**) Test samples.

For rGO-ECC mixtures, cement and fly ash were used as primary and secondary binders, respectively. These are Portland Cement (C) CEM I 42.5 R and F class fly ash (FA). The chemical and physical properties of cement and fly ash are given in Table 1. Silica sand with a maximum grain size of 400 μm was used in ECC mixtures. The distribution of grain sizes of cement, fly ash and silica sand is given in Fig. 1. The cement, fly ash and silica sand are also shown in Fig. 2a. Polyvinyl Alcohol (PVA) fiber, which provides ECC with ductility properties, was used in the mixtures. rGO was added to the ECC mixtures to impart conductivity to ECCs. The geometric, mechanical and technical properties of rGO and PVA fiber are shown in Table 2. 0.0% rGO (Control), 0.4% rGO and 0.6% rGO ratios were selected for the production of rGO-ECC which were calculated as the weight percentage of the total binder amount. The mixing ratios for rGO-ECCs are listed in Table 3. rGOs used in the production of rGO-ECC were weighed with a precision balance and it is shown in Fig. 2b. Since rGO is a nanomaterial, a sonication device was used to prevent agglomeration of its particles and to ensure homogeneous distribution of rGO in the mixture and it is shown in Fig. 2d. Since workability is negatively affected as the amount of rGO increases, the amount of superplasticizer (SP) was used in kilograms for 1 m^3^ fiber concrete mixture amounts as in Table 3. PVA fiber was added and it is shown in Fig. 2c. The materials prepared to produce the test samples shown in Fig. 2g., were mixed with a mixer and they are given in Fig. 2e. The molds into which the mixtures are poured are shown in Fig. 2f. A mini slump flow test was performed to determine the workability properties of fresh rGO-ECCs. As a result of these experiments, workability decreased as the rGO amount increased. In order to control this situation, superplasticizer ratios were used as given in Table 3. The flow diameters obtained from the mini slump flow test are shown in Table 3. As a result of the mini slump flow test, the flow diameter was limited to the range of 34.6 ± 0.6 cm. With the increase in the amount of superplasticizer, the workability properties were similarly controlled for all mixtures.

**Table 1:** Chemical and physical properties of cement and fly ash.

Chemical properties (%)	Fly ash	Cement
CaO	1.70	60.5
SiO_2_	61.6	20.1
Al_2_O_3_	22.1	7.9
Fe_2_O_3_	7.0	3.1
SO_3_	0.1	2.4
MgO	1.7	2.8
Na_2_O	0.3	0.2
K_2_O	2.2	0.8
TiO_2_	0.9	0.3
Physical properties	Fly ash	Cement
Specific gravity(gr/cm^3^)	2.3	3.1
Loss on ıgnition	2.6	2.5

**Table 2:** Geometric, mechanical and technical properties of rGO and PVA fibers.

Fiber type	PVA	rGO
Strength (MPa) (apparent)	1092	-
Strength (MPa) (nominal)	1620	-
Specific gravity(t/m^3^)	1.3	0.25
Length (mm)	8	-
Diameter(μm)	39	1–5
Strain(%)	6	-
Young modulus (GPa)	43	-
Thickness (nm)	-	0.5–2
Specific surface area(m^2^/g)	-	2562
Color	White	Black
Layers	-	3–6

**Table 3:** Mixing ratios and properties for 1 m^3^ rGO-ECC (Kg).

Mixture name	%0.0rGO-ECC	%0.4rGO-ECC	%0.6rGO-ECC
Cement	564	564	564
FA	678	678	678
Water	330	330	330
PVA	26	26	26
Silica sand	451	451	451
SP	5.00	5.45	5.55
rGO	-	4.96	7.44
Flow diameter (cm)	35.2	34.5	34.0

### Compression test

**Fig. 3:**
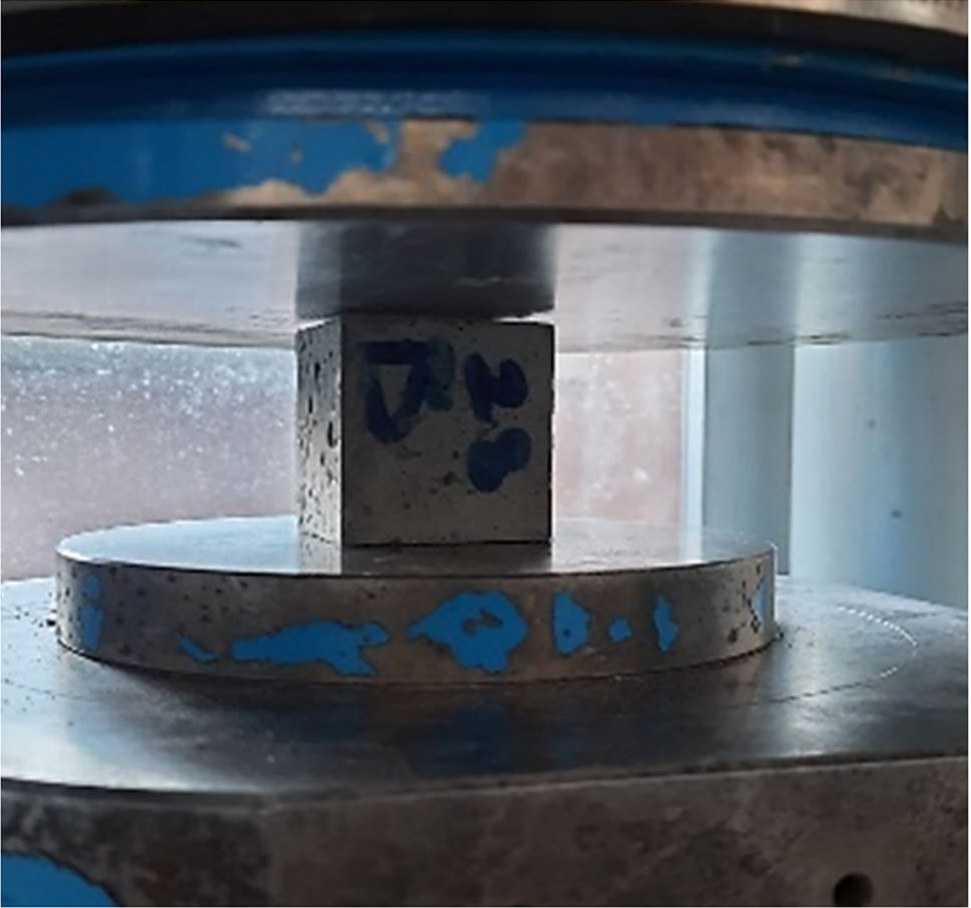
Compression test.

According to ASTM C109^65^, compression test samples of 5 × 5x5cm^3^ dimensions were produced to test the compressive strength values. The samples were subjected to 7 and 28 days of curing in the same environment and conditions in a plastic bag. Compression test was performed on the prepared 5 × 5x5cm^3^ compression samples. The mechanical effects of 0.4% and 0.6% rGO added to the control sample on ECC were investigated. Compression tests were performed with a testing device capable of applying 600kN load as seen in Fig. 3.

### Determination of microstructural properties

XRD, TGA-DTA, FT-IR and SEM–EDX analyses were examined to determine the microstructural properties. The microstructural properties of rGO-ECC blends were analyzed by X-ray diffraction method (XRD), thermogravimetric analysis/differential thermal analysis (TGA/DTA), Fourier Transform Infrared Spectroscopy (FTIR) and Scanning Electron Microscopy with Energy-Dispersive X-ray Spectroscopy (SEM–EDX).

#### X-ray diffraction analysis (XRD)

XRD analysis performed with Panalytical/Empyrean device is based on the principle that each crystal phase breaks X-rays in a characteristic order depending on its unique atomic arrangements. The scanning process of XRD analysis was carried out at 10–90 angular positions [°2θ] and 0.013° steps.

#### Fourier transform infrared spectroscopy (FT-IR)

Infrared (IR) spectroscopy performed with the Bruker/Vertex 70 device; measures the vibration frequencies of various bonds in molecules and provides information about the functional groups in molecules. It is decisive in the structural analysis of functional groups in the structure of organic/inorganic compounds in solid, liquid, gas or solution form. An FTIR spectrum is actually a graph of infrared light transmittance or absorbance as a function of wavelength or frequency. The standard frequency units used in FTIR spectra are wave numbers with the symbol cm^-1^. In FTIR, the analysis of individual spectra detects a chemical reaction in the sample. When infrared light passes through the sample, each functional group causes the spectra to resonate at their characteristic absorption frequencies. The data obtained define the chemical reaction between the molecules. The temperature and relative humidity in the FTIR analysis were kept constant at 20 C° and 60%, respectively.

#### Thermogravimetry and differential thermal analysis (TGA/DTA)

TGA/DTA analysis performed with Linseis/ STA TG-DSC/DTA PT1600 device is called thermogram or thermal decomposition curve, the graph of mass or mass percentage against time, temperature and change in atmosphere. Thanks to this curve, decomposition temperatures and thermal behaviors of materials such as glass, polymer and ceramic are determined. TGA analysis was applied on 18 mg powder samples, in temperature ranges of 25–1000 °C, with a scanning speed of 10 °C/min under nitrogen atmosphere conditions.

#### Scanning electron microscopy with energy-dispersive X-ray spectroscopy (SEM–EDX)

The experiment, which is carried out with the help of a ZEISS Gemini 500 model device, records the morphological structure of the surface with high resolution. Cracks, particle distributions, sizes and pore structure are examined using electron beams. EDX works together with SEM, electron beams excite the atom and emit X-rays specific to the elements. Chemical composition (C, Ca, Si, Al, O and Fe) is determined thanks to the element-specific beams.

### Two-pole conductivity experiment

**Fig. 4:**
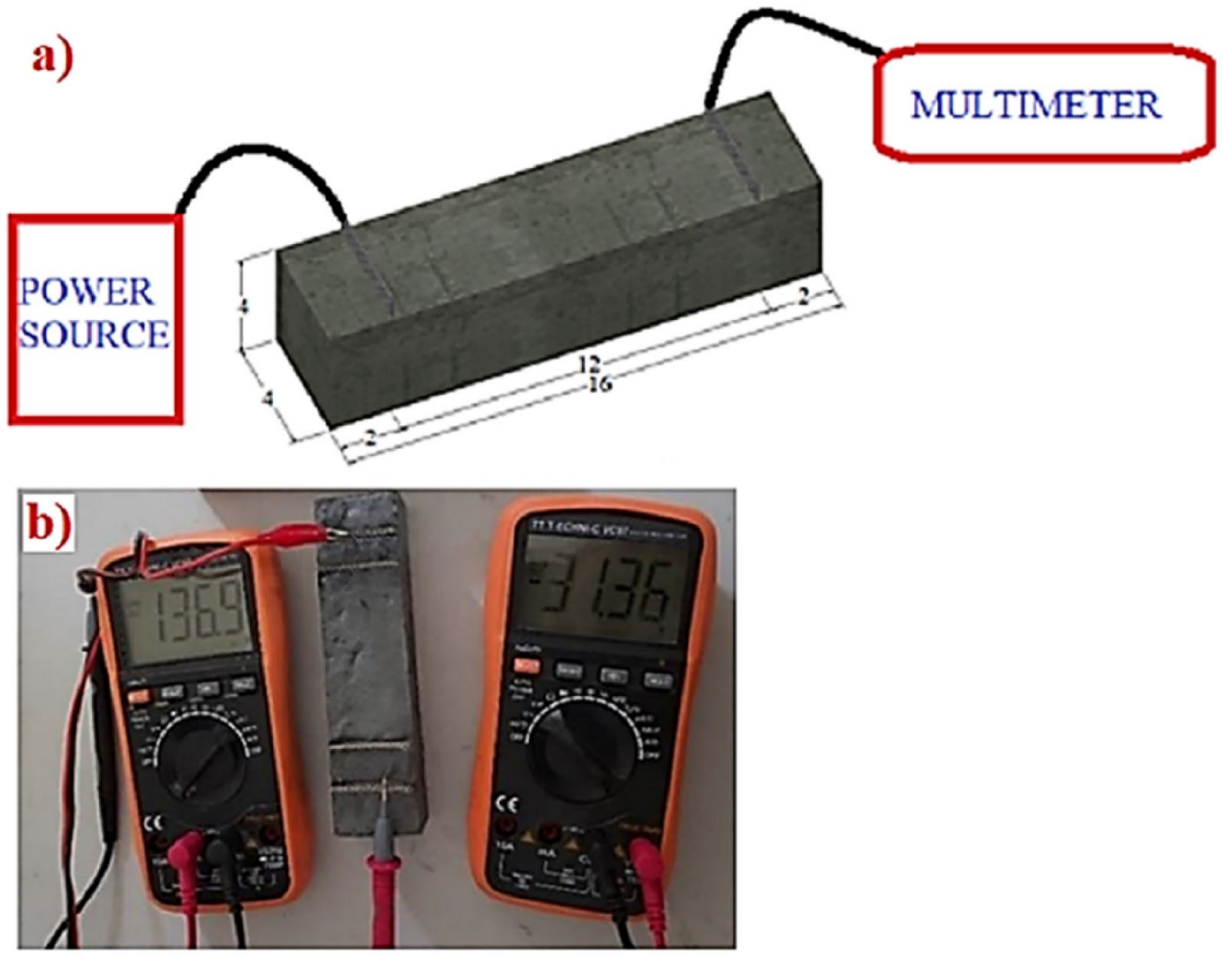
Two-pole conductivity experiment schematic (**a**) and real (**b**).

The two-pole conductivity test is performed by applying electric current from the power source between two poles (probes) placed on the sample and measuring the potential difference between these two probes^66^. The purpose of this test is to determine the resistance, resistivity and conductivity values of rGO-ECCs according to Ohm’s law. Ohm’s law is the voltage obtained by multiplying the current and resistance values. In other words, the product of the ampere and ohm given in Eq. (1) is equal to volt^66,67^. In order to measure the conductivity values, 4 × 4x16cm^3^ samples were prepared. The schematic of the experiment conducted with the samples is shown in Fig. 4a and the actual experimental setup is shown in Fig. 4b. These samples were placed in 38 × 50 mm poles using Type 304 (UNS 30,400) quality stainless steel (steel304) with a chemical composition of 18–20% Chromium and 8–10.5% Nickel in accordance with ASTM A240/A240M-20 (2020)^68^. 304 quality stainless steel was used because it is cheap, durable and has a very low resistance value (1.2 Ω). In order to make resistance calculations of rGO-ECC samples, electric current was given to this 304 quality stainless steel placed as a pole with the help of a power source. The current and volt measurements passing between the two poles were measured with the help of a multimeter capable of measuring at microampere level. These current and volt measurement values were written in the formula (1) given below and resistance calculations were made according to Ohm’s law. In order to calculate the conductivity values of rGO-ECCs, resistivity values must be calculated. The obtained resistance values were written in the formula (2) and the resistivity values were calculated. Finally, it is seen in Eq. (3) that the resistivity and conductivity values are inversely proportional. Moman et al.^61^, Fulham-Lebrasseur et al.^67^, Liu et al.^69^, Dehghanpour et al.^70^ used the two-pole conductivity test in a similar way in their studies and calculations were made with Ohm’s law^71,72^.

1 $$R = \, V/I$$

I: Current (ampere), R: Resistance (Ohm:Ω) and V: Volt^67^.

The resistivity values of rGO-ECC were determined with the help of formula (2) given below for the resistance values found.

2 $$\rho = \left( {R.A} \right)/L$$

*L* Distance between poles, *ρ* Resistivity (Ω.m) and *A* Cross-sectional area^69,70^.

The resistivity values of rGO-ECC are inversely proportional to conductivity as seen in formula (3). Therefore, it can be determined that if the resistivity decreases, the conductivity increases or if the resistivity increases, the conductivity decreases.

3 $$\sigma = 1/\rho$$

σ: Conductivity (1/ Ω.m)^66^.

Direct current (DC) was given to the rGO-ECCs from the power supply and measurements were made at microampere level with the help of a multimeter and the resistance, resistivity and conductivity parameters were determined using the formulas (1), (2) and (3) given above.

### Heating, energy efficiency and ice melting experiments of rGO-ECCs

**Fig. 5:**
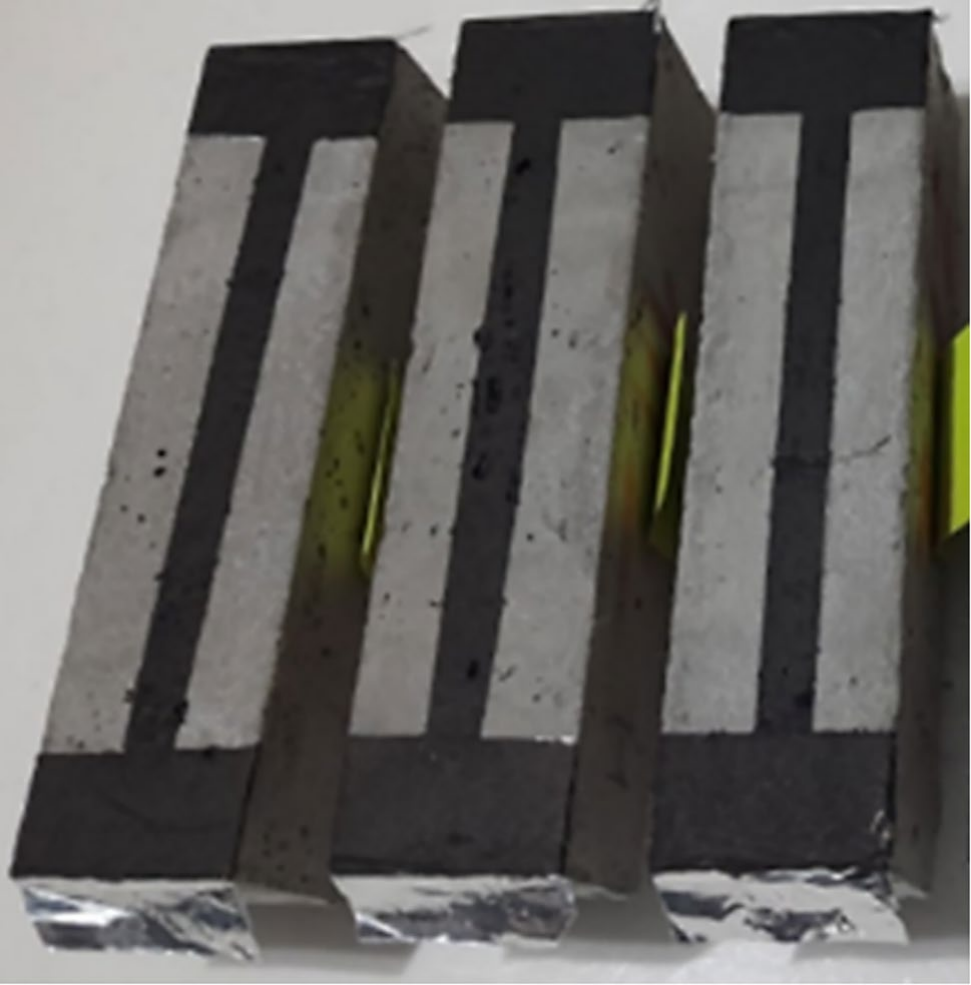
Application of carbon-based conductive coating under heating.

As a result of two-pole conductivity experiments, the conductivity increased while the rGO ratio increases in the mix but did not reach the desired levels. Instead of using more rGO in terms of both cost and mechanical aspects, a more economical and more functional carbon-based conductive coating was applied to the lower surface of the samples and the samples were given electricity to gain self-heating ability and the samples are given in Fig. 5. In order to provide electricity to the coating surface, poles (probes) were made on two sides with aluminum tape and thanks to these aluminum poles, electricity was given to the carbon-based conductive coating and rGO-ECC was heated. A liquid mixture prepared with graphite was used for the production of the carbon-based conductive coating. This coating was applied liquidly to the bottom of the rGO-ECC in the form of a 1 × 12 cm strip and allowed to dry at room temperature (22 C°) for 24 h.

**Fig. 6:**
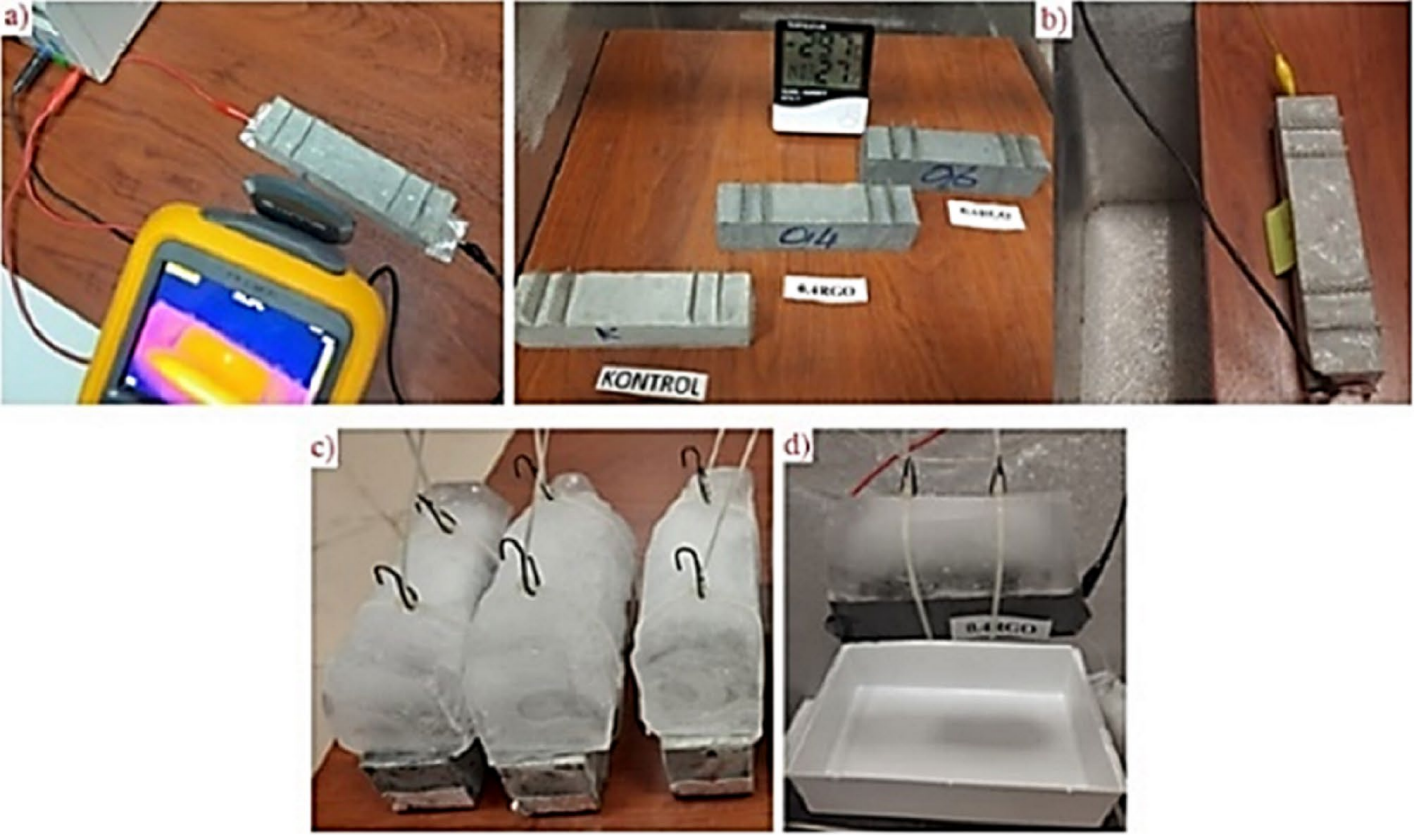
(**a**) Heating in room conditions (22C°), (**b**) Cold environment rehearsal room (-33C°), (**c**) Fixing of artificial ice to rGO-ECC, (**d**) Defrosting in cold environment rehearsal room (-33C°).

In all experiments, a power source that can produce a maximum of 30 V direct current was used for heating the coating. The heating capabilities of rGO-ECCs were examined in three different environments and conditions. The first of the different environments and conditions is room conditions (22 C°), the second is a cold environment rehearsal room (-33 C°) and the third is melting 6 cm of artificial ice in a cold environment rehearsal room (-33 C°). In the studies carried out in the first and second environment conditions, approximately 20 V voltage was applied to heat the samples. Approximately 20 watts (72 kJ) equal amount of power (energy) was applied to the samples heated with the conductive coating. The first environment is shown in Fig. 6a and the second environment is shown in Fig. 6b. In the first and second environments, the average heating rate of rGO-ECC for 60 min (ΔT/minute), energy converted to heat (kJ) and energy efficiency (%) were calculated for heating stability parameters. In the third environment, 6 cm artificial ice fixed on rGO-ECC is seen in Fig. 6c. As the artificial ice will have a continuous cooling effect on the rGO-ECC samples, approximately 25 V of voltage was applied and approximately 31 watts of power was applied until the ice completely melted. The time for the ice to melt completely (minutes), the energy consumed (kJ), the ice melting rate (ml/m^2^.min), the decrease in the amount of ice (%) and the energy efficiency (%) values were calculated. During the experiments carried out in three different environments and conditions, the temperature changes of the samples were measured with a Fluke brand thermal camera at certain time intervals. In this way, the heating stability was tested and the energy efficiency was calculated. With the mechanism set up in the cold environment rehearsal room for the ice to melt, the melted ice dripped onto the scale and the amount of melted ice was determined instantly and the experimental image is given in Fig. 6d.

The data obtained during the heating period in room conditions (22C°) and cold environment rehearsal room (-33C°) were calculated using the formula (4) below.

4 $$Q=MC\Delta T$$

Q: Energy converted to heat, M: Weight of rGO-ECC (Kg), C: Specific heat capacity of rGO-ECC (Joule (J)/Kilogram (kg)*Celsius° (C°)) and ΔT: Temperature change^73^.

The data obtained by melting 6 cm of ice (-33C°) in the cold environment test room was used to calculate the energy efficiency (**η**_**i**_) for the ice melting process using the formula (5) below.

5 $${{\varvec{\eta}}}_{{\varvec{i}}}=({{\varvec{m}}}_{{\varvec{c}}}{{\varvec{C}}}_{{\varvec{p}}{\varvec{c}}}{\boldsymbol{\Delta }{\varvec{T}}}_{{\varvec{c}}}+{{\varvec{m}}}_{{\varvec{i}}}{{\varvec{C}}}_{{\varvec{p}}{\varvec{i}}}{\boldsymbol{\Delta }{\varvec{T}}}_{{\varvec{i}}}+{\boldsymbol{\Delta }}_{{\varvec{s}}{\varvec{o}}{\varvec{l}}}{\varvec{H}}{{\varvec{m}}}_{{\varvec{i}}})/({\varvec{P}}\boldsymbol{\Delta }{\varvec{t}})$$

6 $${\varvec{P}}={\varvec{I}}.{\varvec{V}}$$

7 $${{\varvec{P}}}_{{\varvec{d}}}={\varvec{P}}\boldsymbol{\Delta }{\varvec{t}}/{\varvec{F}}$$

8 $${\boldsymbol{\Delta }{\varvec{T}}}_{{\varvec{A}}}={\boldsymbol{\Delta }{\varvec{T}}}_{{\varvec{c}}}/\boldsymbol{\Delta }{\varvec{t}}$$

9 $${{\varvec{M}}{\varvec{i}}}_{{\varvec{m}}{\varvec{e}}{\varvec{l}}{\varvec{t}}}={{\varvec{m}}}_{{\varvec{i}}}/{\varvec{F}}\boldsymbol{\Delta }{\varvec{t}}$$

P: Power given during melting, Δt: Power given time, m_c_: Weight of rGO-ECC, m_i_: Weight of ice sheet, C_pc_: Specific heat capacity of rGO-ECC, C_pi_: Specific heat capacity of ice, ΔT_c_: Temperature increase of rGO-ECC, ΔT_i_: Temperature change of ice, Δ_sol_H: Heat of fusion of ice, P_d_: Power density, F: Surface area, ΔT_A_: Average heating rate and Mi_melt_: Melting rate of ice^74^.

In this study, Engineering Cementitious Composite (ECC) samples containing reduced graphene oxide (rGO) were prepared. The experimental procedure consisted of multiple steps. First, rGO-ECC mixtures were cast and cured. Then, a series of laboratory tests were conducted, including compressive strength tests, electrical conductivity measurements, microstructural analysis (e.g., SEM–EDX, XRD, FTIR and TGA/DTA), and heating and defrosting experiments under various ambient conditions. In the final stage, measurements were made with an infrared thermal camera to monitor the defrosting behavior and surface temperature responses of the samples during heating. Defrosting times and maximum temperatures were determined. The proposed method enables comprehensive evaluation of the heating and defrosting performance of rGO-ECC by combining pressurized, electrical, and thermal measurements supported by microstructural analysis.

## Results and discussion

### Compression test

**Fig. 7:**
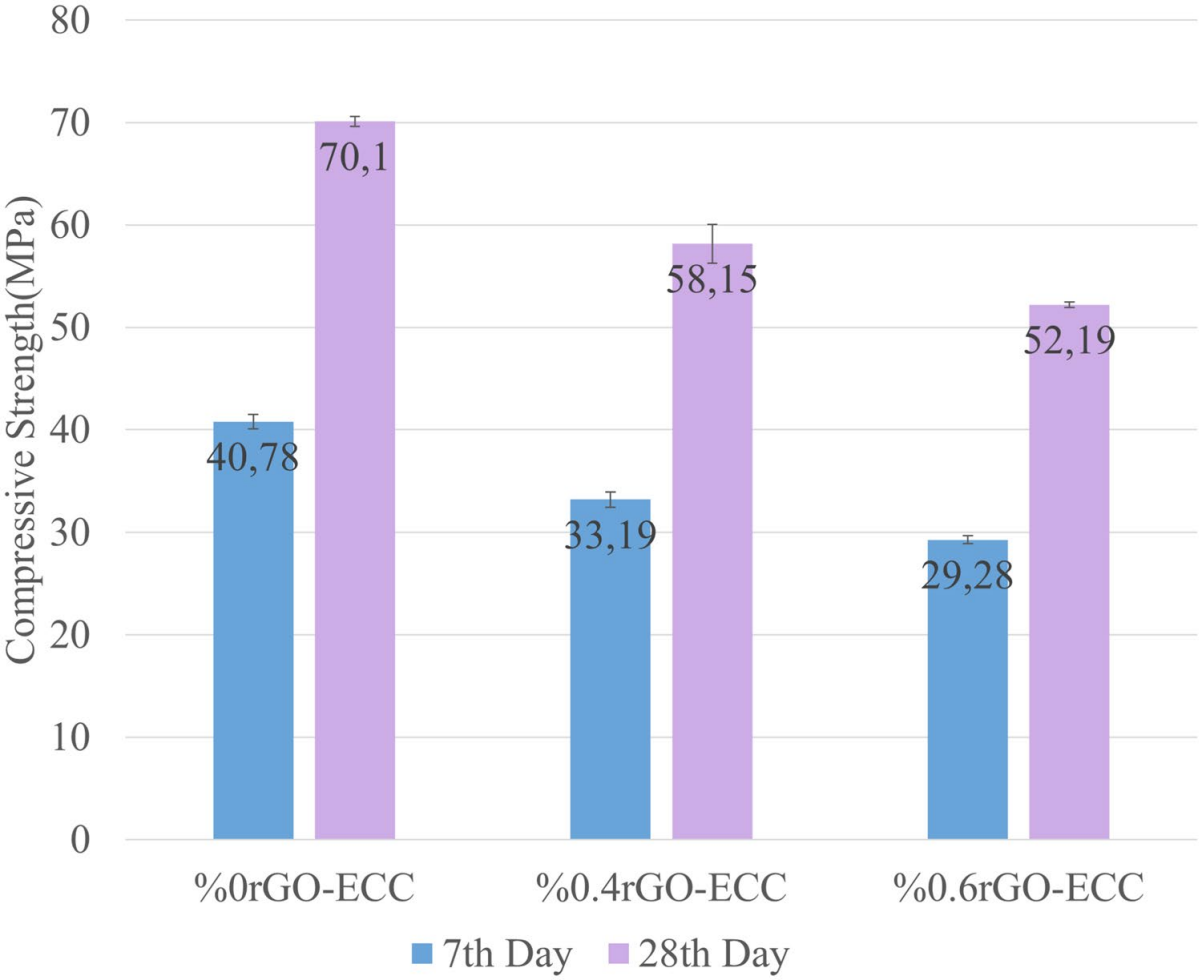
7th and 28th day compressive strength values (MPa).

The compressive strengths of 0.0%rGO-ECC (Control), 0.4%rGO-ECC and 0.6%rGO-ECC samples obtained by compression tests are given in MPa in Fig. 7. When the results are examined, the compressive strength of 0.4%rGO-ECC decreased by approximately 17% and the compressive strength of 0.6%rGO-ECC decreased by approximately 26% compared to the control sample. When the studies using reduced graphene oxide and graphene derivatives are examined, in the study of Ghazanlou et al.^35^, compression tests were carried out using 0.05%, 0.1% and 0.15% rGO and it was reported that the compressive strengths increased by 10%, 25% and 6%, respectively, compared to the control sample, and it was seen that the best result was obtained with 0.1% rGO. When the studies conducted by Madbouly et al.^40^ were examined, it was seen that the rGO ratio that did not negatively affect the pressure values and significantly improved them was 0.05%rGO. In the study conducted by Zhang et al.^41^, it was seen that the rGO ratio that positively improved the compressive strength was 0.08%rGO. It was observed that the mechanical strength values increased by approximately 20% after 28 days of curing by adding 0.1% graphene to the mixtures^75,76^. It was observed that the workability of the mixture using graphene oxide decreased by approximately 35% compared to the control mixture and the best results in the compressive tests were obtained by adding 0.03% graphene oxide^32^. The compressive strength results obtained by adding 0.5%, 1.0%, 1.5% and 2.0% graphene oxide to the control sample were approximately 30 MPa, 42 MPa, 32 MPa, 27 MPa and 25 MPa, respectively, and it was determined that it negatively affected the strength when used more than 0.5% graphene oxide^77^. As a result of the researches, it has been reported in the literature that rGO added composite materials improve their mechanical properties and it has been determined that rGO improves its properties such as compressive strength as a result of using it at optimum values^78^. Reduced graphene oxide is a nanomaterial with a very thin and transparent appearance consisting of two-dimensional carbon atoms in hexagonal form with sp^2^ orbitals and has the ability to fill regular pores (agglomeration) due to this feature^79,80^. It has been determined that the optimum rGO ratio that best fills the gaps in the matrix due to its agglomeration feature and improves the mechanical properties of concrete is between 0.05%-0.1%^32,75–80^. It has been determined that 0.15% rGO used more than the optimum values causes strength loss in the samples^34^. It has been observed that the 0.4% and 0.6% rGO ratios tested showed similar results and caused a decrease in compressive strength values. However, although the use of rGO reduces the compressive strength values of ECC, it has been determined that it remains above the C30/37 MPa limit value, which is the limit value for use in rigid pavements on highways^81,82^.

### Microstructural properties

#### X-ray diffraction analysis (XRD)

**Fig. 8:**
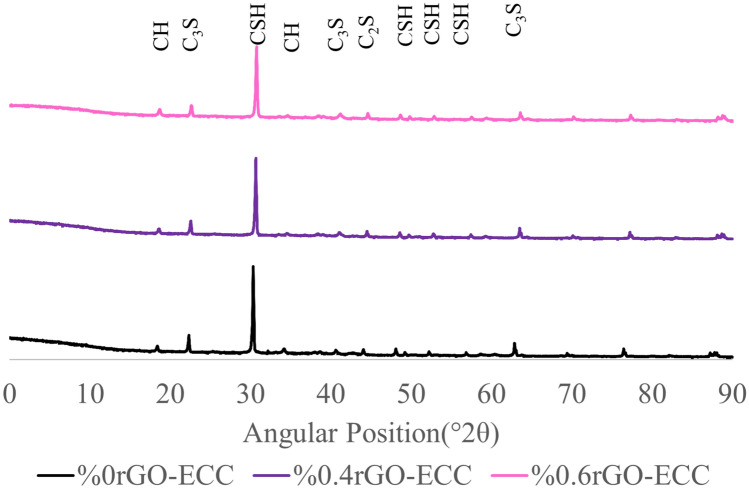
XRD results.

The XRD results of the control (0% rGO-ECC), 0.4% rGO-ECC and 0.6% rGO-ECC samples are normalized for better reading and are given in Fig. 8. In this study, the first peak obtained for the control sample (0% rGO-ECC) and increasing rGO ratios was obtained at approximately 2θ = 23°, and the second peak was obtained at approximately 2θ = 30°. Portlandite (CH) obtained in the first peak, C-S–H gels (tobermorite) obtained in the second peak, and C_3_S (Tricalcium Silicate) and C_2_S (Dicalcium Silicate) formed in the other peaks. When the points of the second peak (2θ = 30°) are examined, it is seen that the intensity of the C-S–H gels decreases as the rGO ratio increases. XRD results showed that there are various mineralogical compounds such as Quartz (SiO_2_-Q), Calcium Silicates, Ca(OH)_2_(CH), C-S–H, CaCO_3_, C_3_S and C_2_S in cement-based composites^83^. It was observed that new crystalline phases were not formed by adding rGO to cementitious mixtures^84^. If rGO is added too much, agglomerated rGO aggregates can be seen and it can cause C-S–H gels to be more irregular and less dense^85^. The changes in Q(Quartz) and CH(Ca(OH)_2_) were investigated for control and increasing rGO ratios. It was observed that Quartz and CH were at close values due to the SiO_2_ content coming from cement, fly ash and silica sand^21^. According to XRD spectra, the first peak of graphene occurred at approximately 2θ = 27° and the d-distance between the layers was measured as approximately 0.3 nm and it was reported that it could be associated with the proper pore filling and agglomeration behavior of graphene^84^. In XRD analysis, the peak of approximately 2θ = 10° and the d-distance of 0.80 nm belonging to graphene oxide was determined as approximately 2θ = 26° and the d-distance of 0.40 nm belonging to rGO after the reduction process^86^. Due to the agglomeration (clumping) feature of rGO used in excessive amounts, the intensity of hydration products at the peaks decreased^84,87^. It was seen that the results obtained from XRD graphs were supported by the compression test.

#### Fourier transform infrared spectroscopy (FT-IR)

**Fig. 9:**
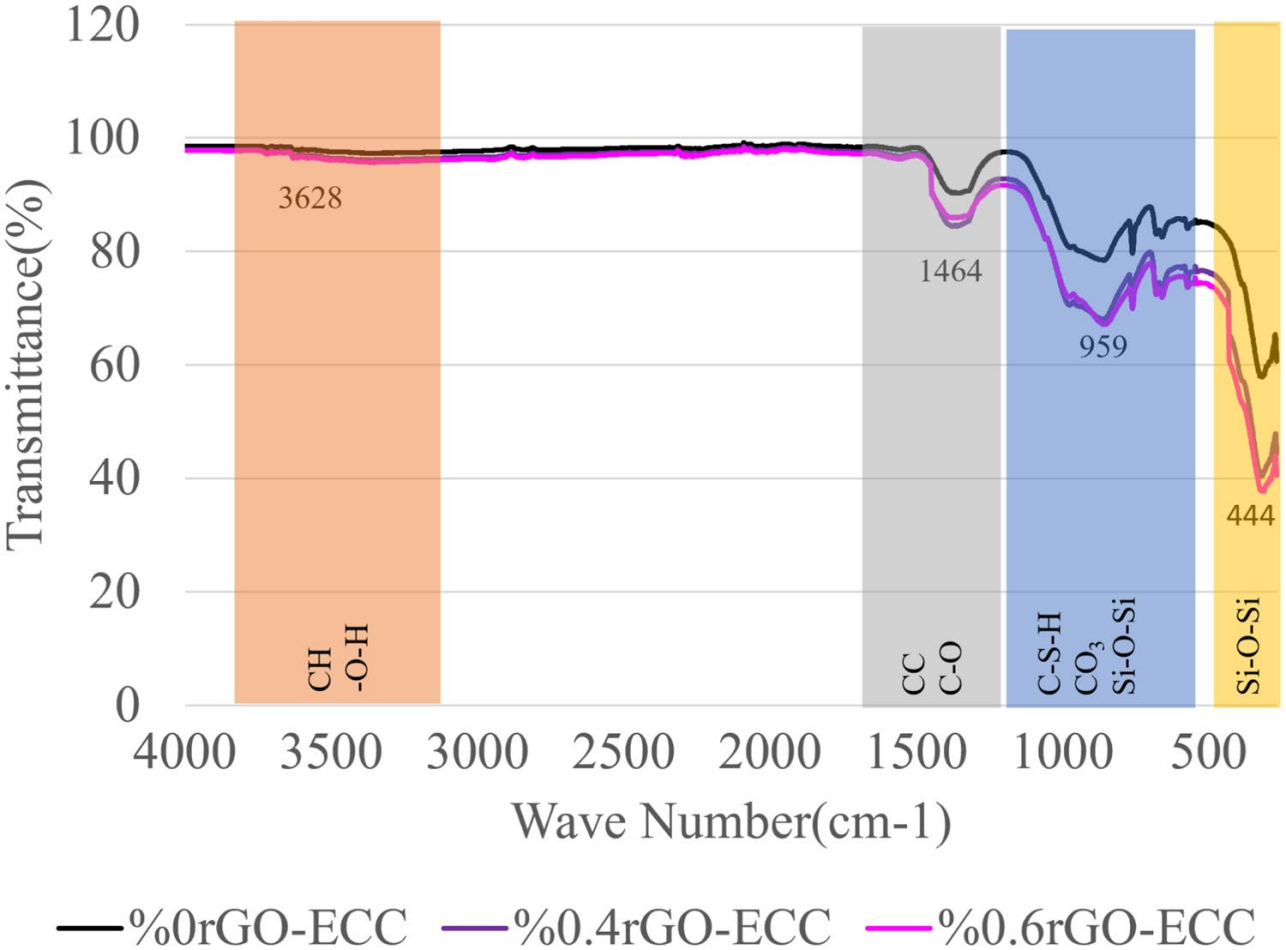
Transmittance graphs of rGO-ECCs.

FT-IR results of control(%0rGO-ECC), 0.4rGO-ECC and 0.6rGO-ECC samples are given in Fig. 9. FT-IR is a function of frequency or wavelength. Additionally, it is known as the peak curve of transmittance or infrared(IR) light absorption^84^. Chemical components of concrete sample can be identified with FT-IR. Unit of FT-IR spectra is cm^-1^ and expressed as wave number^88^. FT-IR analysis was used to identify C-S–H (Calcium Silicate Hydrate), CH (Calcium Hydroxide) (Ca(OH)_2_) and CC (Calcium Carbonate) (CaCO_3_) contents of rGO-ECC. In this study, according to the FT-IR results obtained from Control (0% rGO-ECC), 0.4% rGO-ECC and 0.6% rGO-ECC samples, the peak values obtained at transmittance values at 3628 cm^-1^, 1464 cm^-1^, 959 cm^-1^ and 444 cm^-1^ are shown. According to the FT-IR results, the lower the infrared light transmittance value, the higher the presence of C (CaO) (Calcium Oxide), CH and C-S–H^89^. In other words, if the light does not pass to the opposite side, the C, CH and C-S–H content/density can be interpreted as high. In this study, the peak at 959 cm^-1^ can be mentioned as the presence of C-S–H, CO_3_ and Si–O-Si (Siloxane) structure with asymmetric stretching oscillations (v_3_), which is the vibration mode^90^. In addition, the peak obtained at 444 cm^-1^ can be explained by the presence of Si–O-Si structure with the other vibration mode, which is the bending mode^91,92^. The decrease in C-S–H gel can be explained as occurring due to decalcification^84^. The peaks at wavelength 3628 cm^-1^ indicate the presence of -O–H and CH(Ca(OH)_2_)^90^. In addition, it is seen that there are characteristic C-O and CC(CaCO_3_) peaks at 1464 cm^-1^^89,93^. The primary gel that provides the binding property of concrete is C-S–H and the removal of calcium ions from C-S–H gels is called decalcification. If the CaO/SiO_2_ ratio in C-S–H gel decreases, it causes a decrease in the compressive strength of concrete^94^. According to FT-IR analysis, in the reduced graphene oxide structure, hydroxyl group (-OH) and double bonded carbon (C = C) tensions are observed at 3437 cm^-1^ and 1624 cm^-1^ and functional groups and bond structures belonging to graphene oxide are not observed. It is understood that the bond structures in the graphene oxide structure are degraded by reduction reactions and the hydroxyl group (-OH) and oxygen (-O) groups are removed^86^. In this study, when the Control (0% rGO-ECC), 0.4% rGO-ECC and 0.6% rGO-ECC samples were compared, it was seen that the infrared light transmittance was 58%, 41% and 37%, respectively. It is seen that the light transmittance decreases as the amount of rGO increases. It is thought that this situation is due to the particle and space filling (agglomeration) properties of rGO.

#### Thermogravimetry and differential thermal analysis (TGA/DTA)

**Fig. 10:**
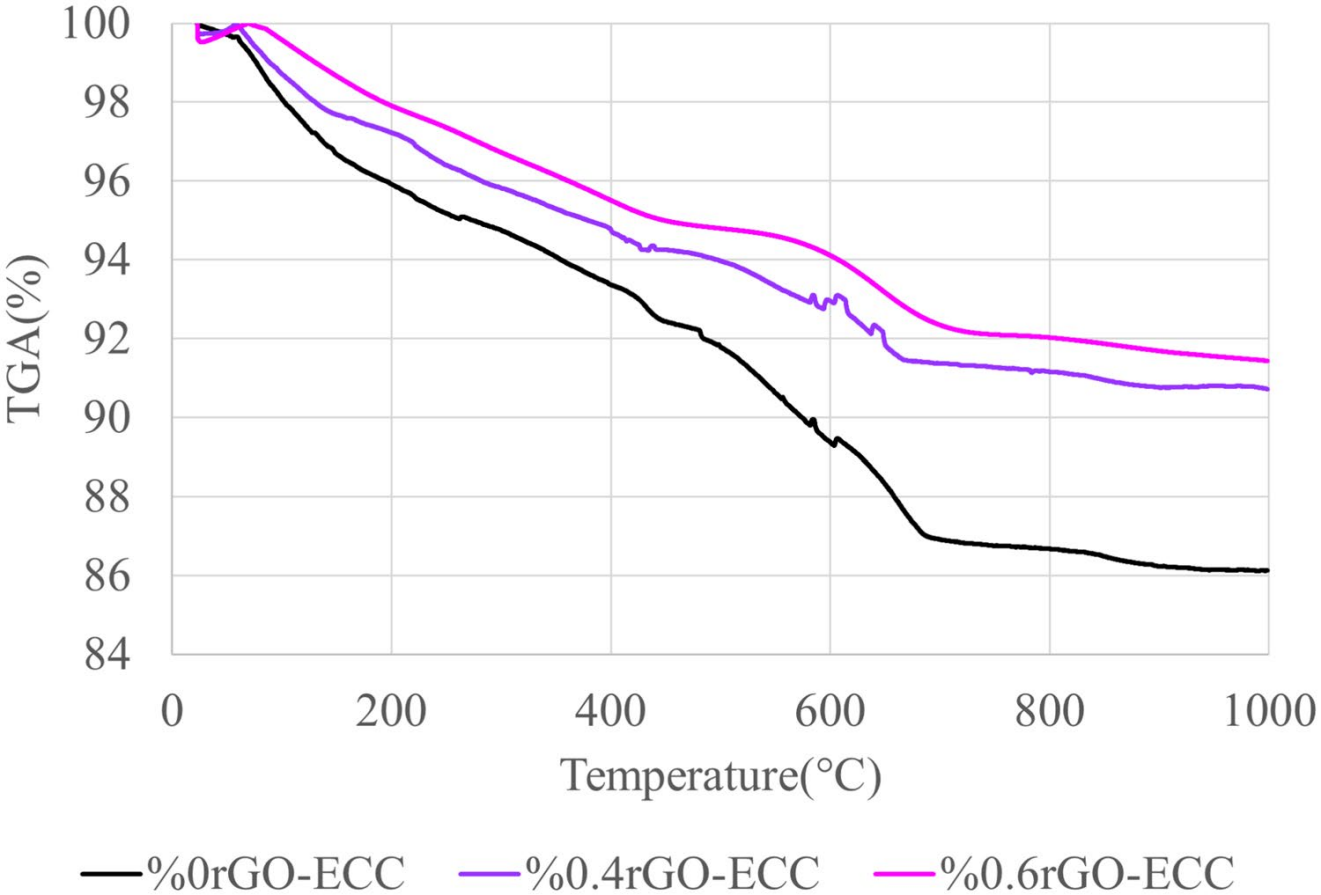
Mass loss graphs of rGO-ECCs.

**Fig. 11:**
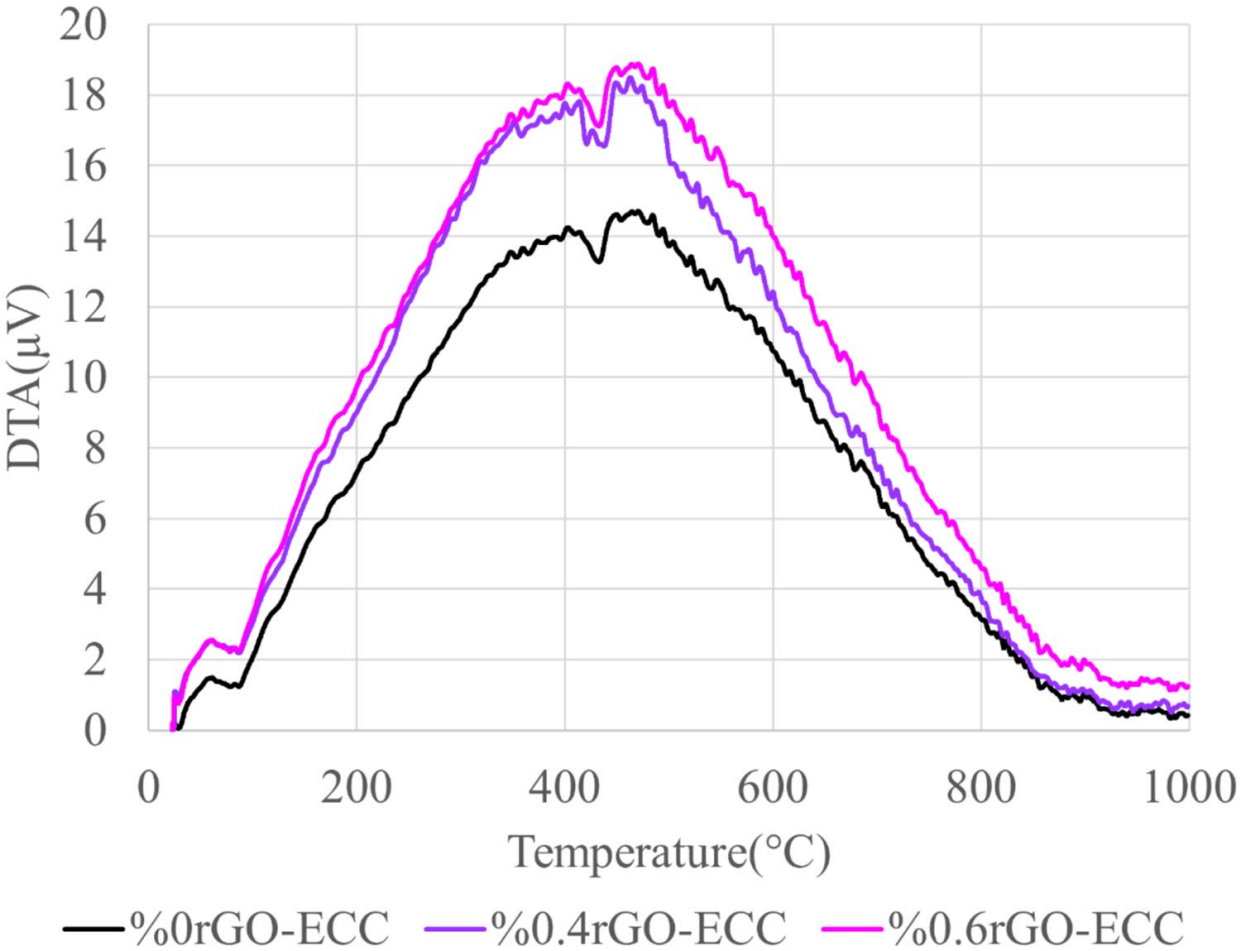
DTA graphs of rGO-ECCs.

The percentage mass loss (TGA) graphs of control (0% rGO-ECC), 0.4% rGO-ECC and 0.6% rGO-ECC samples are given in Fig. 10. In addition, heat flow (DTA) graphs of all ECC mixtures are given in Fig. 11. The TGA/DTA graphs of cement based composites show the dehydroxylation of CH at 425–475 °C and the decarbonization of CC(CaCO3) at 475–765 °C^22^. Moreover, phase changes and transformations in the TGA/DTA diagrams can be observed with the help of peaks. In this study, the TGA/DTA peaks of rGO-ECC at 400–460 °C and 500–725 °C showed the dehydration of CH and the decarbonization of CC, respectively^83^. Therefore, the CH and CC contents in rGO-ECC were determined by the cumulative weight losses determined with the help of TGA graphs in the temperature ranges corresponding to the theoretical weight losses in the DTA curves. The approximate mass loss of the control (0% rGO-ECC), 0.4% rGO-ECC and 0.6% rGO-ECC samples was determined as 14%, 10% and 9%, respectively. It was determined that the mass loss decreased with the increase of the rGO amount^35^. Thanks to rGO, the voids in the microstructure of the concrete are filled more and a denser structure is formed, which reduces the segregation reactions and mass loss^95^. With the addition of rGO, the carbonation resistance in the concrete increases, CaCO_3_ formation decreases and the mass loss observed after 600C°-800C° decreases^96^. With rGO, hydration products are distributed more homogeneously and thermal behaviors improve^97,98^.

#### Scanning electron microscopy with energy-dispersive x-ray spectroscopy (SEM–EDX)

**Fig. 12:**
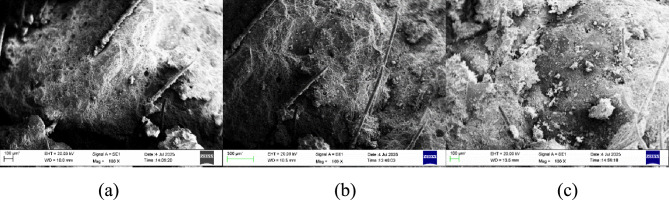
(**a**) 0%rGO-ECC SEM, (**b**) 0.4%rGO-ECC SEM and (**c**) 0.6%rGO-ECC SEM.

**Fig. 13:**
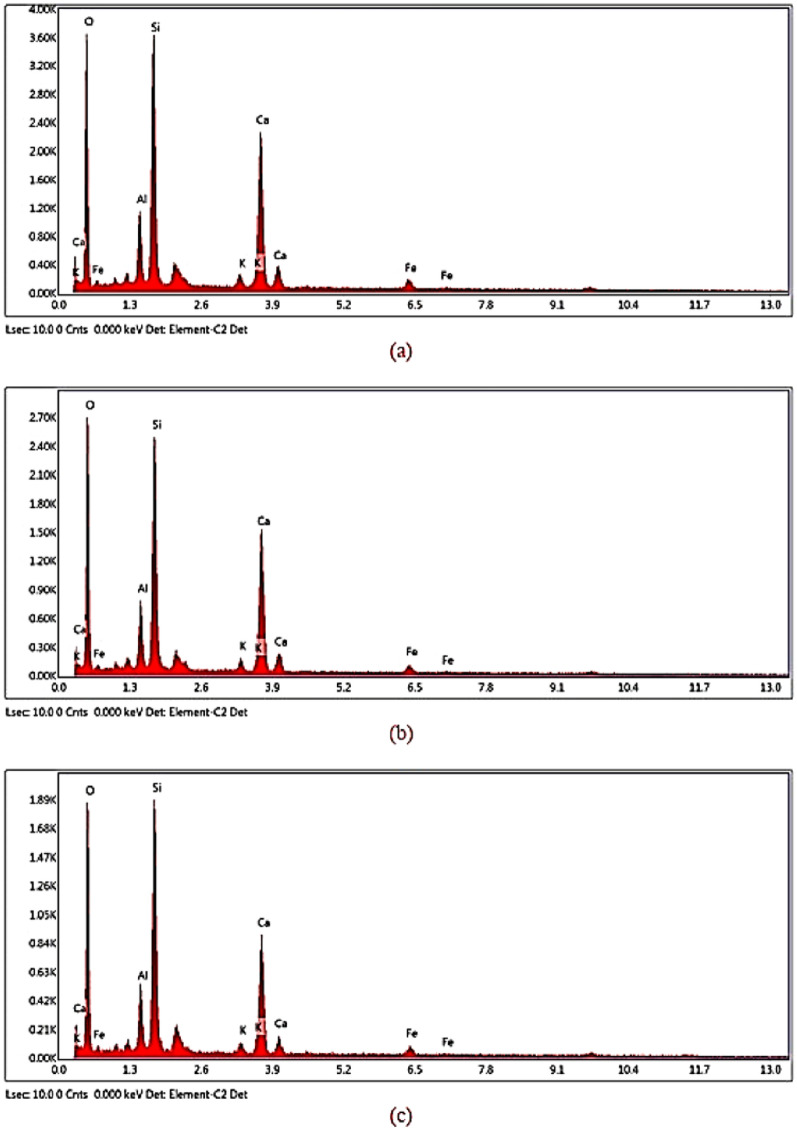
(**a**) 0%rGO-ECC EDX, (**b**) 0.4%rGO-ECC EDX and (**c**) 0.6%rGO-ECC EDX.

The images obtained as a result of SEM–EDX analysis are given in Figs. 12, 13. Chemical composition tables are given in Tables 4, 5 and 6.

**Table 4:** Chemical composition of 0.0%rGO-ECC.

Element	Weight %	Atomic %	Error %
O	55.88	72.48	9.49
Al	5.75	3.66	7.19
Si	19.38	12.84	4.83
K	0.79	0.42	16.00
Ca	17.63	9.64	2.83
Fe	2.57	0.95	18.28

**Table 5:** Chemical composition of 0.4%rGO-ECC.

Element	Weight %	Atomic %	Error %
O	55.98	72.61	9.28
Al	4.57	3.51	6.85
Si	17.32	12.79	4.63
K	0.77	0.41	13.15
Ca	18.66	9.66	2.52
Fe	2.71	1.01	14.46

**Table 6:** Chemical composition of 0.6%rGO-ECC.

Element	Weight %	Atomic %	Error %
O	53.30	70.72	9.47
Al	4.31	3.39	6.81
Si	15.07	12.90	4.52
K	0.78	0.42	13.40
Ca	22.64	11.46	2.50
Fe	2.91	1.11	15.97

SEM images showed that the rGO-ECC matrix was homogeneous and dense. It was observed that the C-S-H gel (tobermorite) formation was in good condition. The hydration products porlandite (CH) and ettringite crystals improved their microstructural integrity with the addition of rGO. PVA fibers are clearly visible. In addition, it was determined that a more compact structure was formed due to the agglomeration feature of rGO.

The meanings of the expressions in the Tables 4, 5 and 6 are; Element: Name of the element being measured, Weight%: Ratio of the element by mass, Atomic%: Ratio of the element according to the number of atoms, Error%: Percentage of error in the measurement. It shows the elements O: Oxygen, Al: Aluminum, Si: Silicon, K: Potassium, Ca: Calcium and Fe: Iron. It is seen that fly ash undergoes significant morphology and chemical composition changes in cementitious composites due to pozzolanic reaction. Fly ash is regular in shape and is close to spherical. Since fly ash has a smooth surface and some foreign components cover it, very reactive fly ash is seen^99^. It is seen with SEM images that fly ash remains in a reactive state. Carbon (C) element could not be detected by the device because the rGO was used in very small amounts. When the peak values of EDX graphics are examined, it is thought that the decrease in strength is caused by the decrease in Si peak values and Si mass which gives an information about C-S–H gels.

### Two-pole conductivity test

**Fig. 14:**
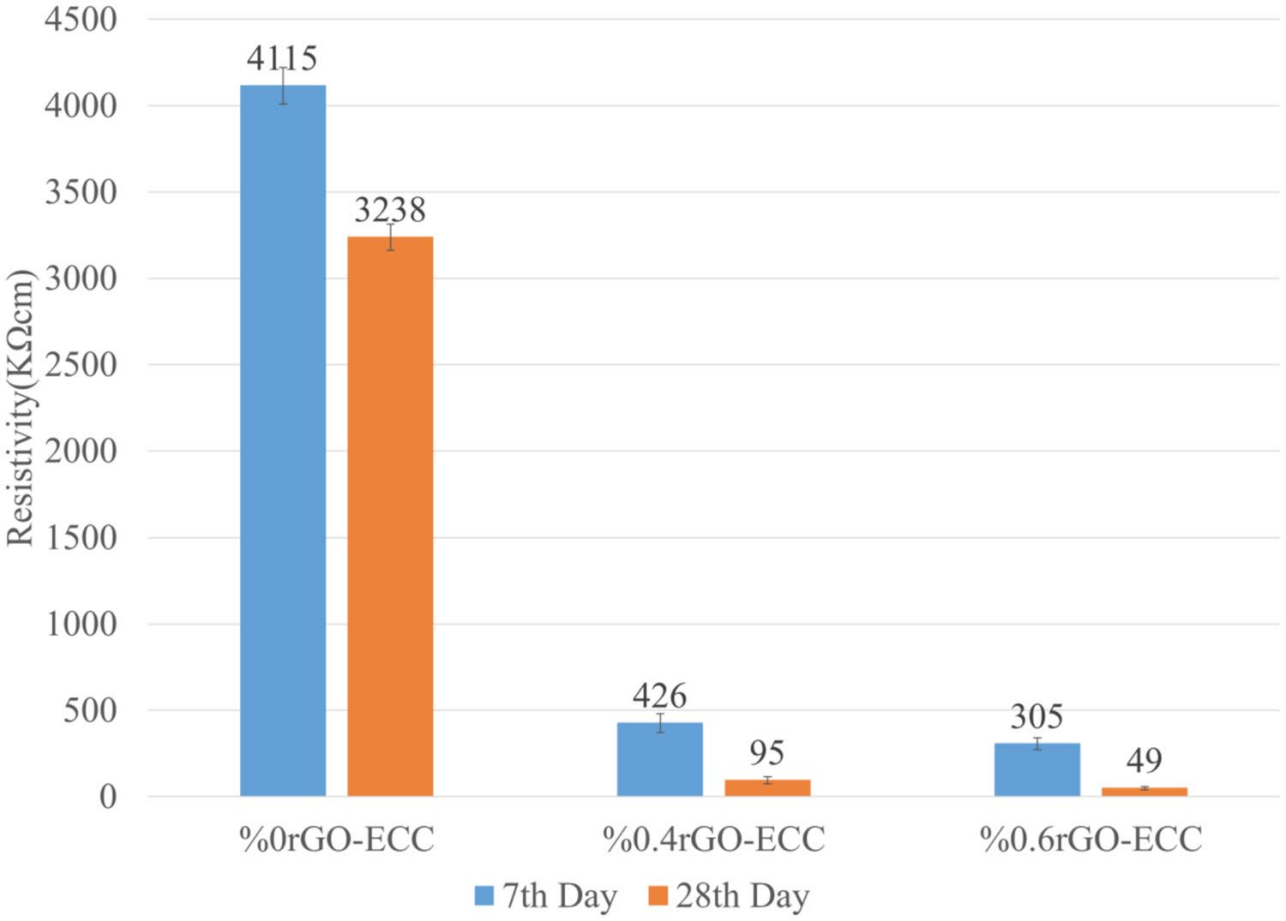
Resistivity values of rGO-ECCs.

As a result of two-pole conductivity tests, the resistivity values of the control (%0rGO-ECC), %0.4rGO-ECC and %0.6rGO-ECC samples obtained on the 7th—28th days of age were measured as 4115KΩ.cm—3238KΩ.cm, 426KΩ.cm—95KΩ.cm and 305KΩ.cm—49KΩ.cm, respectively, with the help of a multimeter. In the study conducted by Sassani et al.^100^, it was observed that the resistivity values of the samples were 115Ω.cm and 992Ω.cm. When the resistivity values of the 7th and 28th days were compared, a decrease was observed in the resistivity value measured on the 28th day. This decrease occurs as a result of the increase in the chemical interaction of C-S–H gels with the completion of hydration, and thus it has been determined that the conductivity increases^80,101^. When the resistivity measurements of 0.6%rGO-ECC mixtures were examined on the 7th and 28th days compared to the control (0%rGO-ECC) sample, it was determined that the rGO became nanolayers formed the electrical conductivity network and the electrical conductivity bond increased with the increase in bond strengths during the curing period. The experimental results are given in KΩ.cm in Fig. 14. The use of rGO in cement mixtures will provide the electrical bond between other materials and will provide the formation of thermally conductive paths^35,69^. Electrical conductivity increases with the addition of rGO to concrete. However, if excessive rGO is used, dispersion problems (such as loss of homogeneity, agglomeration) occur and the increase in conductivity may be limited^102^. rGO added to concrete contributes to electrical conductivity even when used in small amounts by forming a percolation network (conduction network) to conduct electric current^103^. With carbon nanotube (CNT), one of the allotropes of graphene, CNT included cementitious composites were produced for different water/binder ratios. As a result of electrical resistance experiments, it was seen that the mixture with high CNT ratio had high conductivity properties^104^. In another study, it was observed that the electrical resistance decreased with the addition of 0.5% rGO to concrete^102^. The resistivity of the samples with rGO dosage of 0.01%, 0.03%, 0.05%, 0.07% and 0.1% varied between approximately 17KΩ.cm-13KΩ.cm^38^. 0%, 0.05%, 0.5%, 1%, 2% and 4% rGO was added to the concrete and the resistivity decreased between 2.2 × 10^5^Ω.cm-1.2 × 10^5^Ω.cm, and as the resistivity decreased, the electrical conductivity increased^105^.

### Heating, energy efficiency and ice melting performances of rGO-ECCs

#### Heating and energy efficiency of rGO-ECCs at room conditions (22C°)

**Fig. 15:**
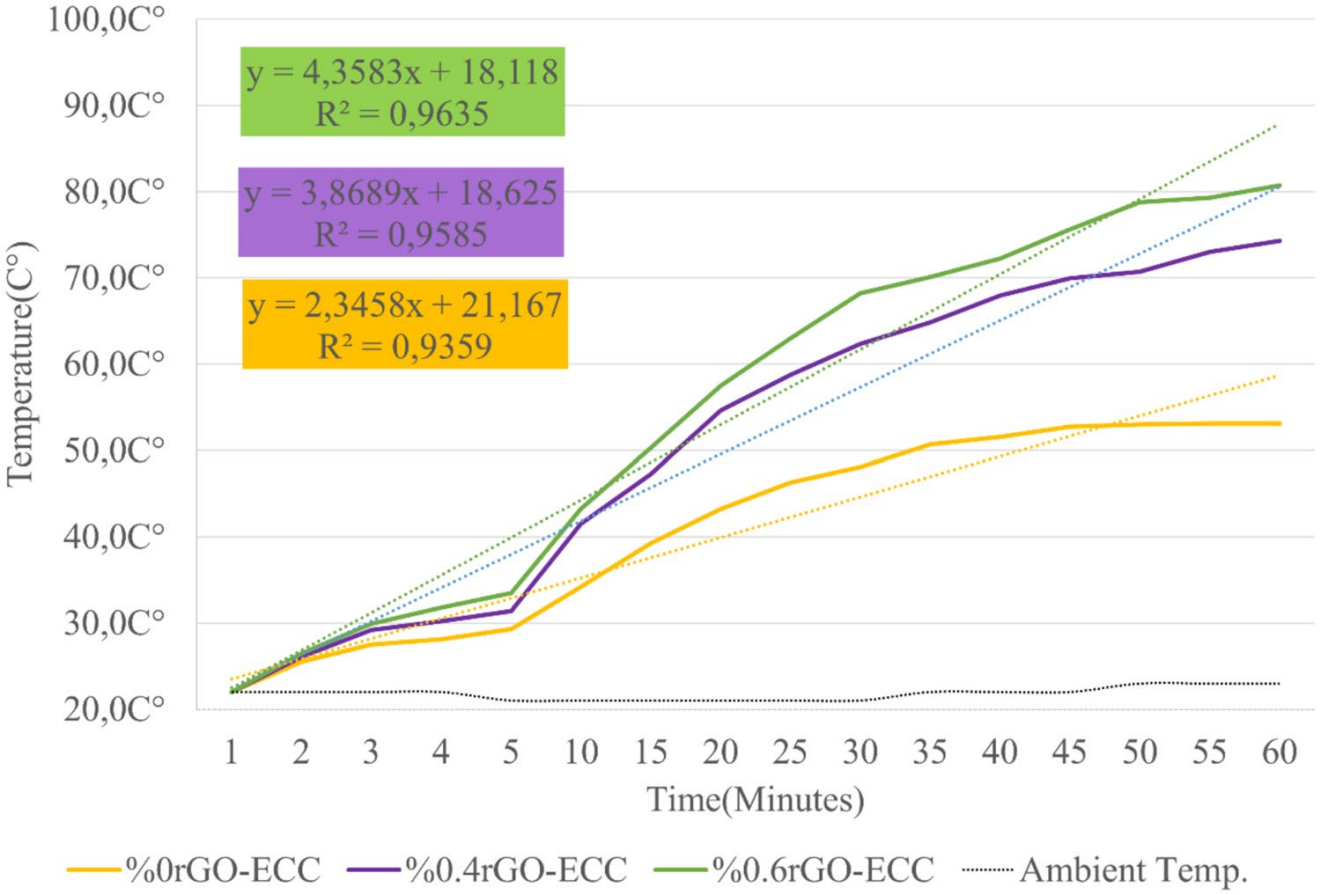
Temperature change amounts obtained in room conditions.

As a result of the experiments, the samples at 22C° reached a maximum of 81C° after 60 min. The temperature changes are given in Fig. 15. The heating parameters of the sample obtained for 60 min were calculated using the Eq. (6) for Power Given (watt), Eq. (7) for Power Density (kWh/m^2^), Eq. (8) for Average Heating Rate (ΔT/minute) and Eq. (4) for Energy Converted to Heat (kJ) and all of these parameters are given in Table 7. The power given at room conditions was kept constant for all sample groups and the situations corresponding to equal energy consumption were examined. The average heating rate was calculated as the heat increase per minute as 0.52, 0.87 and 0.98(ΔT/min.) for Control(%0rGO-ECC), 0.4rGO-ECC and 0.6rGO-ECC, respectively. Compared to the control sample, the temperature increase per minute of the 0.6%rGO-ECC sample was 88.5% higher. When the thermal conductivity coefficients of graphene and concrete were examined, it was seen that the thermal conductivity coefficient of graphene (5300W/mK) was greater than the thermal conductivity coefficient of concrete (0.93W/mK)^106^. In addition, it is thought that graphene and its allotropes added to concrete cause the thermal conductivity coefficient to increase^107^. It was understood that the heat/thermal conductivity coefficient in concrete mixtures depends on the density and the insulation ability decreases with the increase in density^108^. It is thought that rGO added to ECC positively affects the thermal conductivity coefficient of ECC. Therefore, it was observed that the increase in temperature per minute increases positively as rGO is used. In order to determine the amount of energy converted into heat, Eq. (4) was used and it was calculated as 19.3, 35.1 and 43.0 kJ for Control, 0.4%rGO-ECC and 0.6%rGO-ECC mixtures, respectively. Compared to the Control sample, the amount of energy converted into heat in 0.6%rGO-ECC increased by 122.8%. When the results were examined in terms of the rate of conversion of the given energy into heat, namely energy efficiency, it was seen that the Control sample has 26.8%, 0.4%rGO-ECC sample has 48.8% and 0.6%rGO-ECC sample has 59.7% energy efficiency. ECON (Electrically Conductive Concrete) samples produced in 7 × 35x95cm3 dimensions were observed to reach temperatures of from 21 °C, 23 °C and 21 °C to 28 °C, 27 °C and 28 °C in 60 min, respectively^104^. At Des Moines International Airport, when 210 V were applied to the rigid pavement made with ECON concrete, it was observed that ECON reached 30 °C after 400 min from approximately 15 °C^100^. In this study, the samples reached a maximum of 81 °C from room temperature in a short time of 60 min and exhibited a significant heating performance.

**Table 7:** Parameters obtained in room conditions (22C°) for 60 min.

Sample name	Volt (V)	Power given (Watt)	Power density (kWh/m^2^)	Average heating rate (ΔT/min.)	Energy given (kJ)	Energy converted to Heat (kJ)	Energy efficiency (%)
%0rGO-ECC	18.9	20	3.1	0.52	72	19.3	26.8
%0.4rGO-ECC	20.0	20	3.1	0.87	72	35.1	48.8
%0.6rGO-ECC	20.5	20	3.1	0.98	72	43.0	59.7

#### Heating and energy efficiency of rGO-ECCs in cold environment test room (-33C°)

**Fig. 16:**
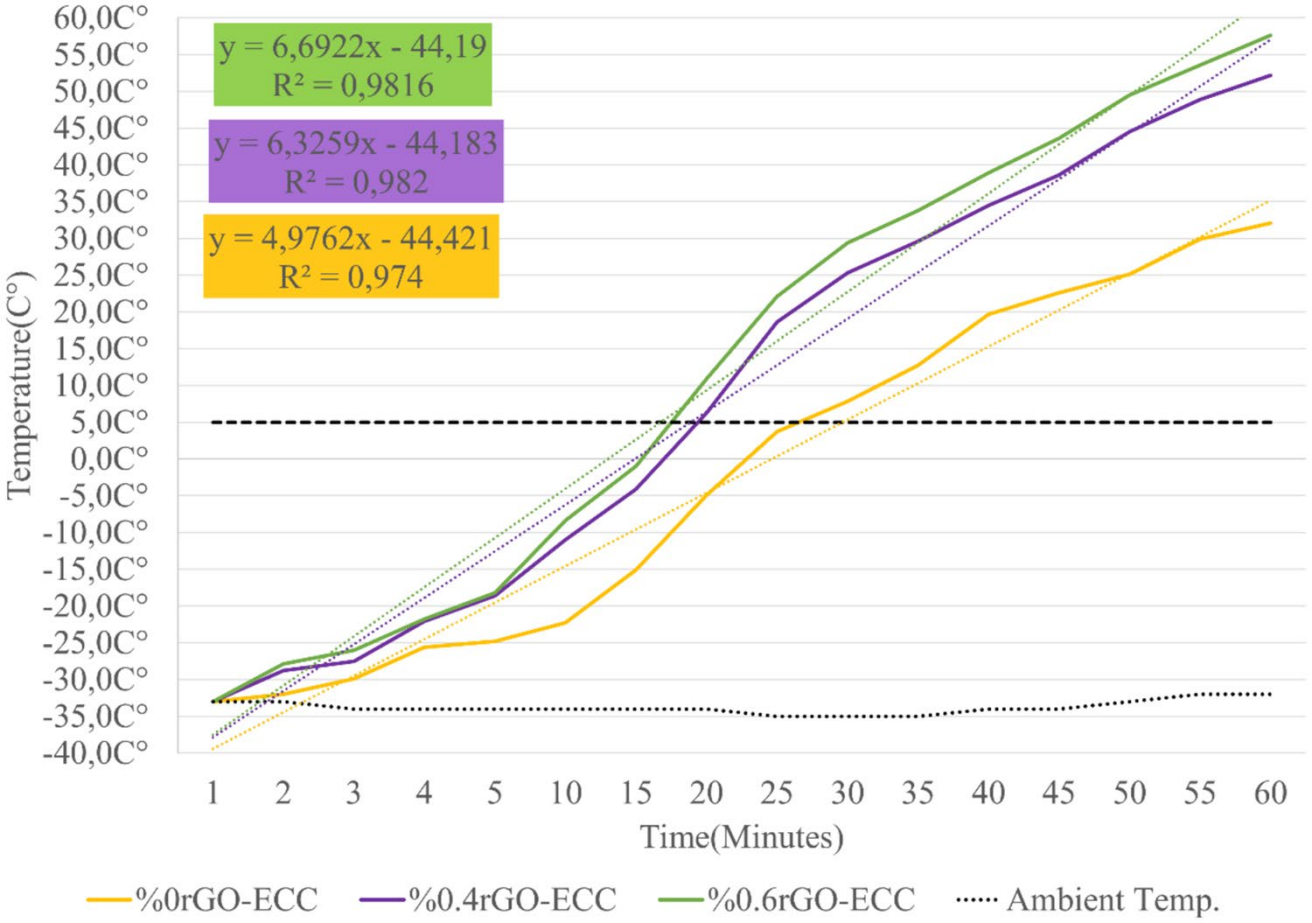
Temperature change amounts obtained in the cold environment test room.

As a result of the experiments, the samples at -33C° reached a maximum of 58C° after 60 min. The temperature changes obtained are given in Fig. 16. The cold environment heating parameters obtained as a result of the calculations are given in Table 8. The power given in the cold environment test room was kept constant for all groups and the situations corresponding to equal energy consumption were examined. The average heating rate (ΔT/minute) was calculated as 1.09, 1.42 and 1.51 (ΔT/min.) heat increase per minute for Control, %0.4rGO-ECC and %0.6rGO-ECC, respectively. Compared to the control sample, the heat increase per minute of the 0.6%rGO-ECC sample was 38.5% higher. It was determined that the heat increase rate in the cold environment was less than in room conditions. It was observed that the environment remaining constantly cold negatively affected the heat increase per minute. It can be concluded that the high heat/thermal conductivity coefficient of rGO significantly positively affects the hot and cold conductivity of rGO-ECC. Formula (4) was used to determine the amount of heat converted to the given energy and was calculated as 43.6, 57.3 and 62.0 kJ for Control, 0.4%rGO-ECC and 0.6%rGO-ECC mixtures, respectively. Compared to the control sample, the amount of energy converted into heat of the 0.6%rGO-ECC sample was 42.2% higher. When the results were examined in terms of the rate of heat conversion of the given energy, namely energy efficiency, it was seen that the Control sample has 60.6%, 0.4%rGO-ECC sample has 79.6% and 0.6%rGO-ECC sample has 86.1% energy efficiency. 40 V voltage was applied to the samples and the samples heated for 25 min reached a temperature between -25C° and 0-5C°^43^. In the study conducted by Sassani et al.^73^, it was reported that samples that were approximately -5C° were heated for 90 min and reached a maximum of 25C°. In this study, experiments were carried out in a cold environment for 60 min and the temperature values increased from -33C° to 58C° and the energy efficiency was 86.1%. With these results, it was seen that rGO-ECC reached high temperatures in a short time with low energy consumption.

**Table 8:** Parameters obtained in the cold environment test room (-33C°) for 60 min.

Sample name	Volt (V)	Power given (Watt)	Power density (kWh/m^2^)	Average heating rate (ΔT/min.)	Energy given (kJ)	Energy converted to heat (kJ)	Energy efficiency (%)
%0rGO-ECC	18.9	20	3.1	1.09	72	43.6	60.6
%0.4rGO-ECC	20.0	20	3.1	1.42	72	57.3	79.6
%0.6rGO-ECC	20.5	20	3.1	1.51	72	62.0	86.1

#### Ice melting ability of rGO-ECCs and other heating parameters

**Fig. 17:**
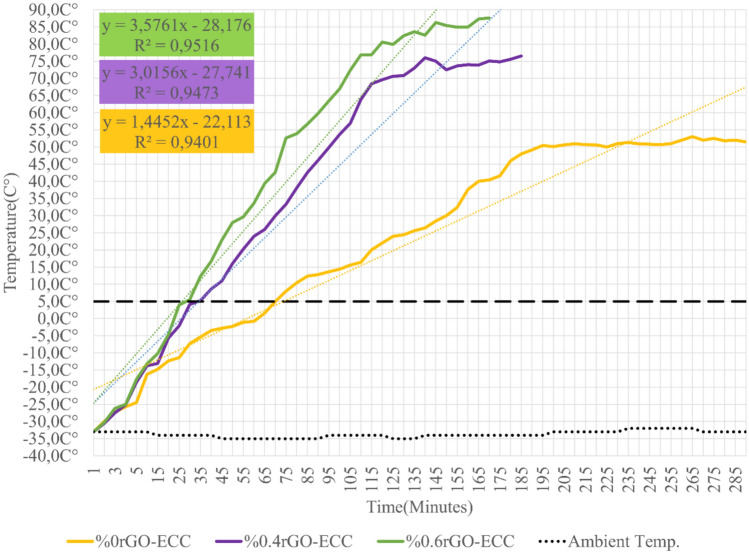
Temperature change amounts as a result of melting ice in a cold environment.

**Fig. 18:**
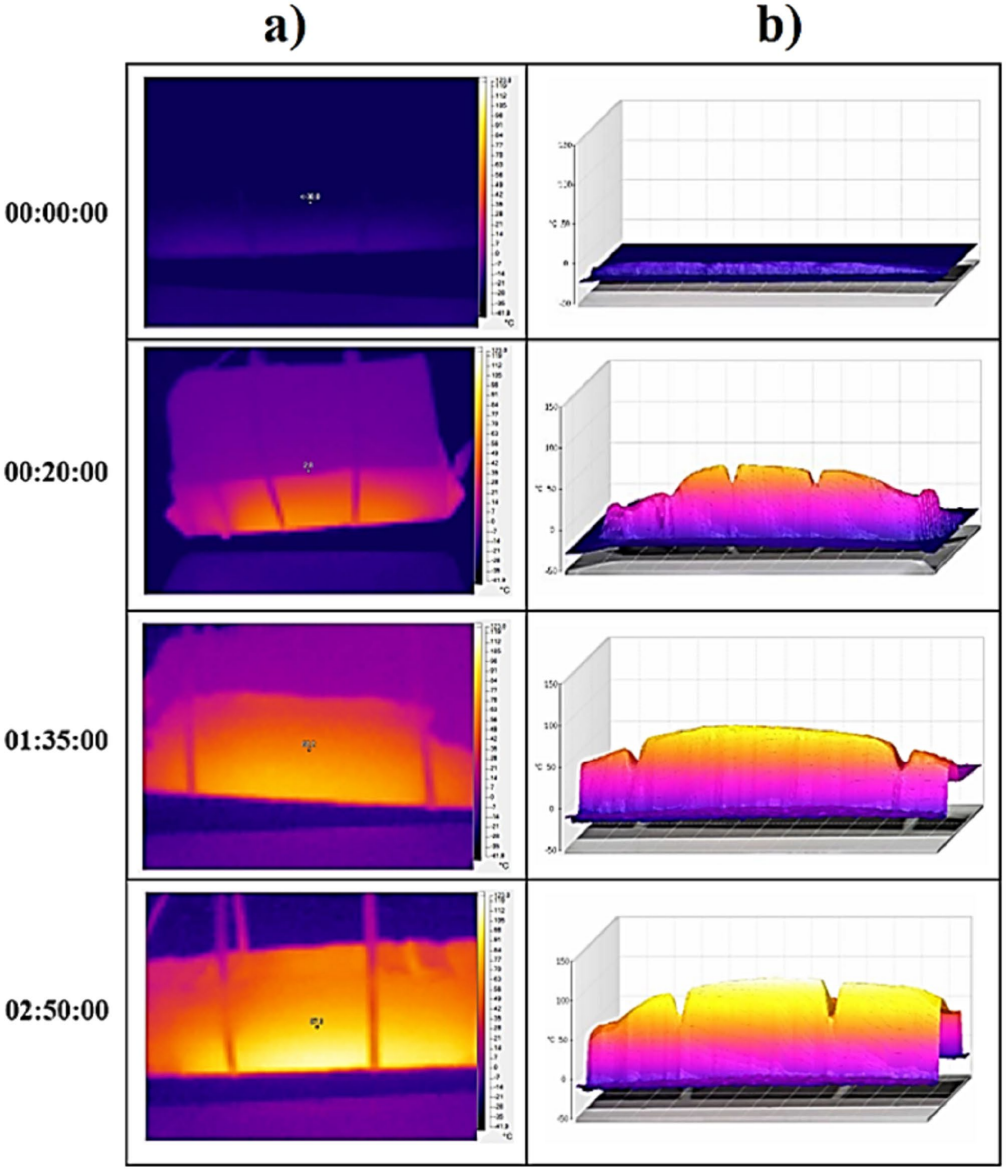
0.6%rGO-ECC (**a**) Thermal camera images obtained during ice melting (**b**) Three-dimensional infrared (3D-IR) thermograms obtained from thermal camera images.

**Fig. 19:**
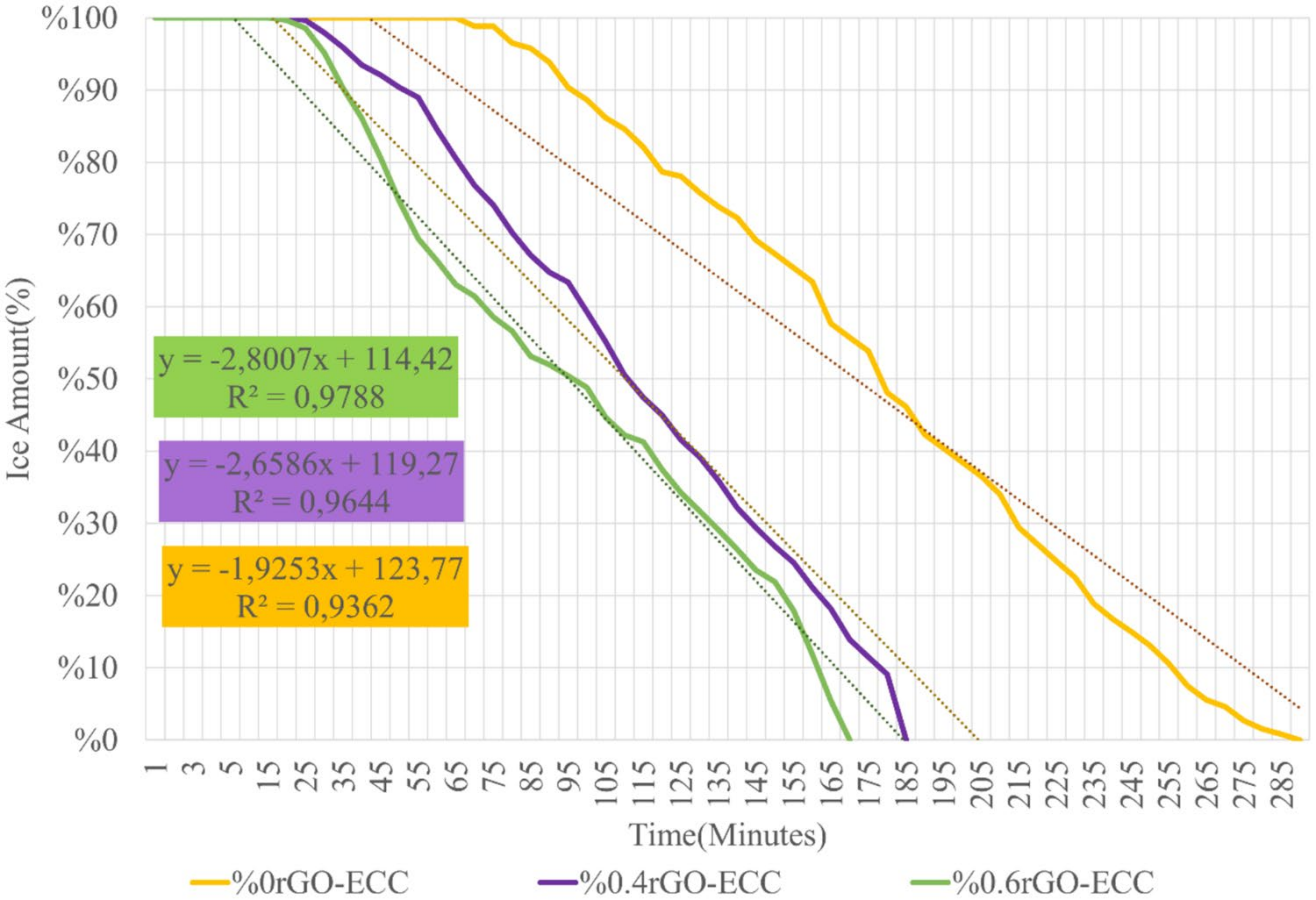
Decrease in the amount of ice during the experiment (%).

**Fig. 20:**
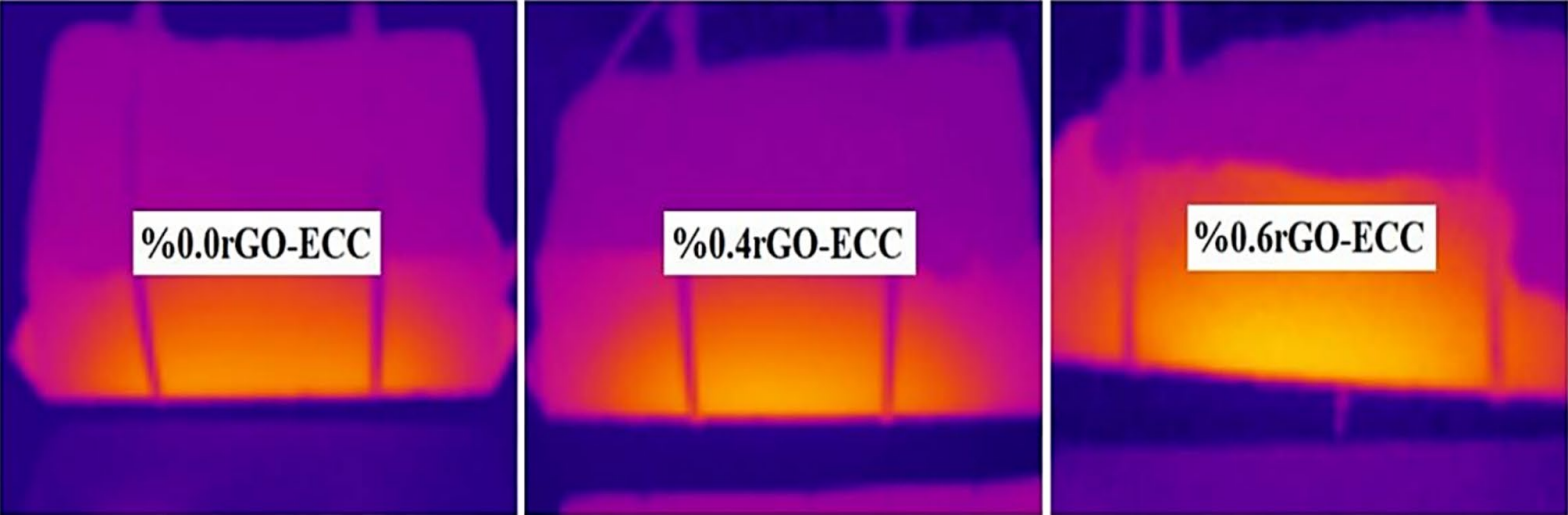
100 min after the start of the ice melting.

The temperature changes obtained as a result of the experiments are given in Fig. 17. The cold environment heating parameters obtained as a result of the calculations are given in Table 9. The power given to the samples to melt the ice in the cold environment rehearsal room was kept equal, but since the ice melting time (Δt) was different in each sample, the total energy given throughout the experiment was calculated as 546.1, 346.9 and 317.3 kJ for Control, 0.4%rGO-ECC and 0.6%rGO-ECC, respectively. The average heating rate (ΔT/minute) was calculated as 0.29, 0.59 and 0.71 (ΔT/min.) heat increase per minute for Control, 0.4%rGO-ECC and 0.6%rGO-ECC, respectively. Compared to the control sample, the heat increase per minute of the 0.6%rGO-ECC sample increased by 144.8%. The negative effects of cold environment and ice were prevented by increasing the applied power from 20 to 31 watts. The power application time of the experiment was reduced by increasing the average heating rate value. In addition to the room and cold environment conditions, the ice melting rate parameter was calculated with the help of Eq. (9). The ice melting rate values were calculated as approximately 179, 293 and 325 ml/m^2^.minute for control, 0.4%rGO-ECC and 0.6%rGO-ECC, respectively. In order to determine the amount of energy converted to heat, formula (5) was used and the energy converted to heat (kJ) was calculated. It was determined as 157.8, 183.3 and 200.3 kJ for control, 0.4%rGO-ECC and 0.6%rGO-ECC mixtures, respectively. Compared to the control sample, the amount of energy converted to heat in 0.6%rGO-ECC increased by 26.9%. The rate of conversion of given energy into heat, namely energy efficiency, was calculated with Eq. (5) and it was seen that the energy efficiency was 28.9% for the control sample, 52.8% for 0.4%rGO-ECC and 63.1% for 0.6%rGO-ECC. According to the results obtained in the cold environment, it was seen that the amount of energy decreased during the ice melting process. Two important points were determined here. The first is that the ice constantly tries to cool the sample during the experiment. The second is the height of the ice layer. The thicker this layer is, the colder the environment will remain. In both cases, it directly affects the energy efficiency. Ice prevents the heating of the sample and reduces the energy efficiency. It was determined that as the ice layer thickness increases, the energy efficiency decreases, and as the ice layer thickness decreases, the energy efficiency increases. During the experiment, temperature increases were recorded with a Fluke brand thermal camera at certain periods. Thermal camera images obtained from 0.6%rGO-ECC at 00:00:00, 00:20:00, 01:35:00 and 02:50:00 min are given in Fig. 18a. and three-dimensional infrared (3D-IR) thermograms obtained from thermal camera images are given in Fig. 18b. Thermal camera images and 3D-IR thermograms are given with a color scale. Black, blue and purple indicate that the samples are cold. Red, orange, yellow and white indicate that the samples are hot. In addition, the amounts of ice melted with the increase in temperature were weighed and recorded with the help of the scale under the experimental platform at the same time periods and are given in Fig. 19. The time taken for the ice to melt completely decreased with the addition of rGO. As a result of the ice melting experiments, the control (0% rGO-ECC), 0.4% rGO-ECC and 0.6% rGO-ECC samples completely melted the ice after 290, 185 and 170 min, respectively. The maximum temperature increased to 51.5 °C, 76.5 °C and 87.6 °C, respectively. With the use of 0.6% rGO, ice melting was completed in 120 min shorter than the control sample and energy was saved. In Fig. 20., thermal camera images of 0% rGO-ECC, 0.4% rGO-ECC and 0.6% rGO-ECC samples after 01:40:00 min (100 min) are given. The ice melting performances of the samples are seen more clearly with the thermal images given after the same period of time after the experiment started. The temperature increase of 0.6% rGO-ECC was 70.1% more than the control sample. In the study conducted by Rahman et al.^109^ for ECON concrete, the temperature of 13.75 × 60x60cm^3^ samples increased to 16C° in 90 min. The temperature of 4 × 4x16cm^3^ beam samples increased to approximately 50C° in 18 min with ECON concrete. The best temperature increase rate was determined to be approximately 0.22C°/minute. Snow mass was melted with rigid pavement made at DSM International Airport using ECON concrete^110^. Sassani et al. ^73^ applied 81.20 V voltage to melt 6 cm of ice in their study. Ice melting energy efficiency was measured as 66%. It was observed that the samples increased from approximately -5C° to 25C° in 90 min. Ding et al.^74^ completely melted 9 mm artificial ice, 5 cm naturally accumulated snow and 2 cm artificially compacted ice in their study. In the samples to which 30 V voltage was applied, the energy efficiency was measured as 99.96% at the highest and 55.44% at the lowest. The temperature change increased from 20 °C to 82 °C in 30 min and in addition, it was observed that the ice thickness directly affected the energy efficiency^74^. When the results of the experiments were compared, it was seen that rGO-ECC provided advantages in parameters such as energy efficiency and ice melting rate.

**Table 9:** Heating parameters determined by completely melting ice.

Sample name	Volt (V)	Power given (Watt)	Power density (kWh/m^2^)	Average heating rate (ΔT/min)	Energy given (kJ)	Ice melting rate (ml/m^2^.min)	Energy efficiency (%)
%0rGO-ECC	23.9	31	23.7	0.29	546.1	179	28.9
%0.4rGO-ECC	25.0	31	15.1	0.59	346.9	293	52.8
%0.6rGO-ECC	25.5	31	13.8	0.71	317.3	325	63.1

### Cost analysis

With construction and building material prices being quite high today, it is important that the prices of concrete produced are economical. Due to the scarcity and importance of financial resources, the studies conducted need to be examined from an economic perspective. Therefore, a comparison of ECC and rGO-ECC was made in terms of cost. The cost analysis studies conducted were made on an average basis based on retail sales in Turkey in 2024 and converted to dollars with the current dollar exchange rate. Transportation, depreciation, labor, operating profit and expenses were not taken into account. Only material prices were determined as a result of the market research conducted. Unit prices and materials obtained in retail are given in Table 10.

**Table 10:** Materials and unit prices (kg/m^3^).

Material name	ECC	Price($)	rGO-ECC	Price($)
Cement(Kg)	565	75	565	75
Coarse aggregate(Kg)	0	0	0	0
Fine aggregate(kg)	451	90	451	90
Fly ash(kg)	678	150	678	150
Super plasticizer(liter)	5	50	5	50
PVA fiber(Kg)	26	1170	26	1170
rGO(Kg)	0	0	1,25	2500
Total		1535$		4035$

As a result of the cost analysis, it was seen that the prices of rGO-ECC were higher than ECC. In their study in Australia, Gholampour et al.^84^ reported that graphene and its derivatives would bring an additional cost of $80–300 per kilogram to concrete. In their analysis, they calculated the cost of graphene as $190. However, they reported that this high cost would become suitable for the construction sector with the development of technology and the increase in production volume^111,112^. The Economic Index (EI) parameter, which is the ratio of strength values to production costs for rGO-ECC, was calculated. The most suitable results for the EI value are obtained when the strength is high and the cost is low. The 7-day EI was calculated as the highest 0.027 and the lowest 0.0073. The 28-day EI was calculated as the highest 0.046 and the lowest 0.013. It was determined that the EI value increased with age^113^. In Turkey, graphene and its derivatives can be supplied by very few companies and the production process in a laboratory environment is costly and time-consuming. The developing technology and the increase in the number of manufacturing companies will make the prices of graphene and its allotropes economical in Turkey and will make its use widespread in concrete mixtures. With the use of affordable graphene in concrete production, ecological concrete production can be made that will contribute to the insufficient natural resources in nature. With the use of graphene and its allotropes in rigid road pavement, accidents resulting from icing will be prevented. Additionally, material and moral losses will be prevented and a direct contribution will be made to the country’s economy.

### Environmental benefits

When producing concrete, it is important to add waste materials and use less cement for environmental policies and to protect natural resources. With these methods, the damage caused by the construction sector to nature can be reduced. The production of nature-friendly and green concretes can only be achieved in this way. Both environmentally friendly and economical concretes can be produced by using more waste materials and less cement. For example, the final price of steel fiber reinforced self-compacting concrete (SFRSCC) exceeds the price of conventional concrete due to its higher cement content, the use of superplasticizers and the addition of steel fibers. Therefore, using waste ceramics reduces construction costs and contributes to nature due to its low price^113^. Copper slag (CS) aggregate, a waste material, was used instead of normal aggregate at 0%, 10%, 20%, 30%, 40%, 50% and 60% for the production of environmentally friendly roller compacted concrete (RCC). Using 60% copper slag provided a 47.18% reduction in concrete production costs. Using RCCs containing copper slag (CS) waste will provide significant economic savings and benefits to nature. In this way, waste copper slag storage costs and damage to nature are reduced^114^. The addition of reduced graphene oxide (rGO) to engineering cementitious composites (ECC) offers significant environmental advantages, especially in cold weather, for melting ice. The rGO-ECC, which becomes conductive, can heat up and melt ice when connected to a power source. It does not cause the damage caused by chemical de-icers. It reduces soil and water pollution and minimizes infrastructure damage. In addition, with the increase in the amount of rGO, it melts ice in a shorter time and provides energy efficiency. With the use of rGO-ECC, a longer service life and less waste material are contributed to nature. In addition, since traffic accidents caused by icing will be eliminated, pollution of nature after accidents (accident waste and more exhaust fumes) is prevented. By using optimum rGO in rGO-ECC, a decrease in the amount of cement is achieved. By using less cement, it contributes to natural resources and environment. rGO-ECC minimizes the environmental impact related to construction. rGO-ECC supports sustainable, durable, environmentally friendly, long-term economical and smart infrastructure solutions. rGO-ECC contributes to the elimination of environmental problems because it is a multifunctional ECC. Firstly, it has superior strength performance and limited crack formation. Maintenance-repair requirement is less. In this way, waste material formation, natural resource consumption and maintenance costs are less. Secondly, thanks to the improved thermal and electrical conductivity of rGO-ECC, it does not harm the infrastructure and nature such as chemical de-icers and salting. Thirdly, by using the optimum rGO ratio in rGO-ECC production, compressive strength is increased. This can reduce the amount of cement. It contributes to natural resources and the environmental damage caused by cement production is reduced. Fourthly, traffic accidents will be prevented on roads with ice in winter months and the environmental damage of waste and emissions that will occur after the accident will be prevented. Finally, the multifunctional structure of rGO-ECC supports the development of smart, energy-efficient infrastructure. Therefore, it contributes to the reduction of energy demand and carbon footprint.

## Summary and conclusions

In this study, an innovative concrete pavement with both high ductility and heat resistance was produced to address ductility and icing problems in rigid pavements. The mechanical properties of rGO-ECCs, as well as their electrical and thermal properties in various environments, were investigated. By adding rGO to ECC, conductivity was increased, and the resulting rGO-ECC was used to address the icing problem on highways. The results obtained from this study are presented below.

- Since rGO was added more than the optimum amount, the compressive strength decreased by 26%. However, it was seen that it was above the technical specification limit value. It would be advantageous to use the produced rGO-ECC as a pavement overlay.
- Microstructural experiments show that as the amount of rGO increases, the decrease in compressive strength is supported by the decrease in C-S–H density. The presence of CH, -O–H, CC, C-O, Si–O-Si, C-S–H, CO3 and Si–O-Si structures was observed. The agglomeration feature of rGO caused the infrared (IR) permeability to decrease. With rGO, the micro-sized voids of the concrete were filled more and a denser structure was formed.
- It was observed that with the increase in the amount of rGO, the resistivity values decreased and an electrical conductivity network was formed.
- rGO-ECCs consumed less energy and achieved an energy efficiency of 59.7% in room conditions (22C°) and 86.1% in cold conditions (-33C°). In the ice melting test, energy efficiency was 63.1%. In the ice melting test in a cold environment (-33C°), it was determined that ice continuously cooled the sample and the thickness of the ice layer negatively affected the results. It was determined that ice melting performance increased with the increase in the amount of rGO.
- It has been observed that the production costs of rGO-ECC are higher than ECC. The economic index supports this result. However, the high ductility provided by ECC to the pavement overlay will reduce operating costs throughout the life of the structure and will become more economical in the long term with its contribution to nature. With the mechanical and conductive properties of the multifunctional rGO-ECC, ice will not form in busy intersections, tunnels and roads in winter months and accidents will be prevented.
- As a result of this study, the deficiencies of ECC were eliminated with the addition of rGO. An environmentally friendly concrete that contributes to the economy and nature in the long term was produced. It was observed that using the optimum amount of rGO would contribute to the strength and economic index. It was determined that using rGO-ECC as a solution to icing problems on highways would prevent material and moral losses. In future studies, increasing the amount of rGO and applying it on larger data sets for validation purposes can be considered. Moreover, different tests regarding highway pavement overlay performances can also be tried.

## Data Availability

The datasets used and/or analysed during the current study available from the corresponding author on reasonable request.

## Abbreviations

ECC: Engineered cementitious composites
rGO: Reduced graphene oxide
rGO-ECC: Enhanced ECC with rGO
PVA: Polyvinyl alcohol
GO: Graphene oxide
XRD: X-ray diffraction
FTIR: Fourier transform infrared spectroscopy
TGA-DTA: Thermogravimetric analysis
SEM–EDX: Scanning electron microscopy with energy-dispersive x-ray spectroscopy
C/CEM I: Portland cement type I
FA: Fly ash
SS: Silica sand
3D-IR: Three dimensional infrared temperature graph
